# Regional heterogeneity of the blood-brain barrier

**DOI:** 10.1038/s41467-025-61841-8

**Published:** 2025-08-08

**Authors:** Marie Blanchette, Kaja Bajc, Benjamin D. Gastfriend, Caterina P. Profaci, Nadine Ruderisch, Cayce E. Dorrier, Guo Zhong, Raquel Cuevas-Diaz Duran, Sean S. Harvey, Iris H. Garcia-Pak, Lucija Pintarić, Manon Leclerc, Louise Reveret, Vincent Émond, Annette Wang, Deepti Pant, Linus T. Tsai, Frédéric Calon, Nina Isoherranen, Sean P. Palecek, Eric V. Shusta, Jiaqian Wu, Richard Daneman

**Affiliations:** 1 Departments of Pharmacology and Neurosciences, University of California, San Diego, La Jolla, CA USA; 2 Department of Chemical and Biological Engineering, University of Wisconsin–Madison, Madison, WI USA; 3 AbbVie, Cambridge, MA USA; 4 Department of Pharmaceutics, School of Pharmacy, University of Washington, Seattle, WA USA; 5 Tecnologico de Monterrey, Escuela de Medicina y Ciencias de la Salud, Monterrey, N.L Mexico; 6 Axe Neurosciences, Centre de recherche du CHU de Québec-Université Laval, Québec, QC Canada; 7 Faculté de Pharmacie, Université Laval, Québec, QC Canada; 8 Department of Medicine, Harvard Medical School, Boston, MA USA; 9 Department of Neurological Surgery, University of Wisconsin–Madison, Madison, WI USA; 10 Department of Neurosurgery & Center for Stem Cell and Regenerative Medicine, UTHealth, Houston, TX USA

**Keywords:** Blood-brain barrier, Neuro-vascular interactions

## Abstract

The blood-brain barrier (BBB), formed by specialized endothelial cells (ECs), regulates the extracellular composition of the central nervous system (CNS). Little is known about whether there are regional specializations of the BBB that may control the function of specific neural circuits. We use single cell RNA-seq to characterize ECs from nine CNS regions in male mice: cortex, hippocampus, cerebellum, spinal cord, striatum, thalamus, hypothalamus, midbrain, and medulla/pons. Although there is a core BBB transcriptional profile, there are significant regional specializations. Stra6, a retinoid transporter, is highly enriched in the BBB of the nucleus accumbens shell (ShNAc) and ventral cochlear nucleus, and is controlled by dietary vitamin A, through endothelial RARƔ. EC Stra6 regulates the deposition of retinoids specifically in the ShNAc and cochlear nucleus, and is required for the function of the ShNAc, in a retinoid-dependent manner. Thus regional specializations of the BBB can regulate the function of local brain regions.

## Abstract (web summary)

The blood-brain barrier (BBB) regulates the extracellular composition of the central nervous system (CNS), but it is not known whether its properties differ across CNS regions. Here, the authors show in mice that the BBB exhibits regional specializations, and that such specializations can be important for the function of specific neural circuits.

## Introduction

The BBB is a term used to describe the unique properties possessed by CNS blood microvessels that tightly control the composition of the CNS extracellular environment, which is essential to maintain CNS homeostasis and protect the neural cells from potentially harmful blood-borne toxins and pathogens^1,2^. Many of these BBB properties are possessed by the endothelial cells (ECs), which line the walls of CNS vessels. These properties include tight junctions, which create a paracellular barrier, lack of fenestra and slow rates of transcytosis, which create a transcellular barrier, as well as unique transport, metabolic, and signalling programs. Together, these properties tightly regulate the movement of ions, molecules, and cells between the blood and the brain^1,2^.

Each region of the CNS performs a unique function and is composed of highly specialized neuronal and glial cell populations. While it is clear that there is heterogeneity of ECs between different organs as well as among different branches of the vascular tree^3–9^, much less is known about whether there is heterogeneity of the BBB among different brain regions. The circumventricular organs, such as the area postrema, subfornical organ, pineal gland and median eminence, have leaky fenestrated vessels that lack BBB properties^10^. These regions require contact with the blood for either sensory or secretory purposes, and thus, the lack of a BBB is critical for the function of the local circuitry. There is, however, much less known about whether there are specializations of the BBB in brain regions that contain a functional BBB, including the cortex, hippocampus, cerebellum (Cb), spinal cord (SC), striatum, thalamus, hypothalamus, midbrain, medulla, and pons. Regional specializations in the physical, transport, metabolic, signaling, or immune properties could differentially control the local environment, which could regulate the development, function and/or plasticity of specific neural circuits. Further, proteins expressed on the luminal EC surface in specific brain regions may provide ‘signposts’ to guide localized drug delivery.

In this work, we used single-cell RNA-seq (scRNA-seq) to profile the transcriptomes of ECs acutely isolated from nine CNS regions: cortex, hippocampus, Cb, SC, striatum, thalamus, hypothalamus, midbrain, and medulla/pons. We found that although the primary heterogeneity observed in these data reflects arterial/capillary/venous zonation, there are a number of robust regional differences in BBB gene expression. These regionally enriched and depleted genes include transporters, immune modulators, transcriptional regulators, and signalling factors. To understand functional implications of this regional heterogeneity, we analyzed the stratium-enriched gene *Stra6*, which encodes a transmembrane retinol (ROL) transporter^11–13^. On the protein level, Stra6 is highly enriched in the ECs of the rostral medial shell of the nucleus accumbens (rmShNAc) and the posterior ventral cochlear nucleus (PVCN), and expression in these regions is controlled by dietary vitamin A and endothelial RARƔ. Using EC-specific Stra6 conditional knockout mice, we found that EC Stra6 is required to deliver retinoids to the rmShNAc and PVCN, but not other brain regions. We also found that EC Stra6 is required for spatial memory, which is mediated by the ShNAc, but not for behaviors mediated by adjacent brain regions. Together, these results establish regional heterogeneity of the BBB as a factor in controlling local extracellular environment and neural circuit function.

## Results

### Transcriptional heterogeneity of the BBB across regions

We first performed bulk RNA sequencing (RNA-seq) on ECs acutely purified from three large regions of the mouse CNS: the forebrain (Fb), Cb, and SC, as well as the liver and lung (Supplementary Fig. 1, Supplementary Data 1). These brain regions greatly differ in the proportion of white matter to grey matter, with the mouse Fb having minimal white matter, whereas the Cb and SC each have significant white matter. As expected, there was a high correlation of gene expression between ECs isolated from the different CNS regions, as each expressed a core set of BBB-enriched transcripts. We did, however, identify significant regional heterogeneity, including 217, 370, and 288 brain EC transcripts enriched in the Fb, Cb, and SC, respectively (Supplementary Fig. 1B, C). GO analysis identified enrichment of sulfur compound catalysis and vitamin transport in Fb ECs, mannosylation and histone deubiquitination in cerebellar ECs and cytokine signalling in SC ECs (Supplementary Fig. 1D). These data suggest that there is a core BBB-specific transcriptional profile throughout the CNS, but there are indeed regional specializations of CNS ECs across large CNS regions.

**Fig. 1:**
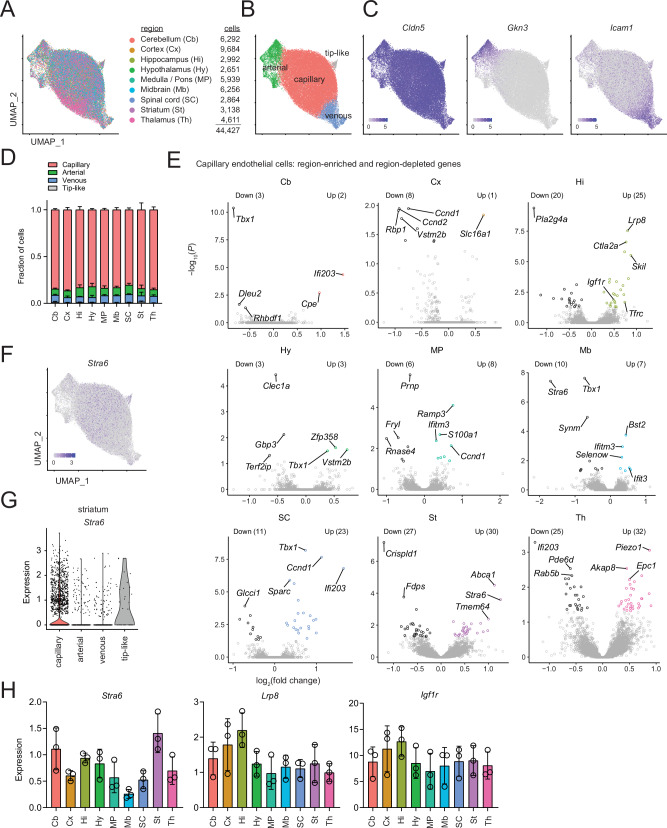
scRNA-seq of endothelial cells from nine brain regions. **A** UMAP plot of all endothelial cells. Plotted points (cells) are colored by brain region from which they were isolated. The number of endothelial cells analyzed from each brain region is shown at right. Each region includes three biological replicates (independently isolated and sequenced cells pooled from 3–6 mice). **B** UMAP plot with points colored by clusters representing endothelial arteriovenous subtypes. Clustering strategy is shown in Supplementary Fig. 2. **C** UMAP plots with points colored based on expression of indicated genes. *Cldn5* (pan-BBB endothelial), *Gkn3* (arterial-enriched), and *Icam1* (venous-enriched) are shown. Color bars indicate expression (log-normalized counts). **D** Fraction of capillary, arterial, venous, and tip-like endothelial cells across brain regions. Bars indicate the mean of three biological replicates and error bars indicate standard deviation. Fraction arterial endothelial cells across brain regions: *F*(8,18) = 1.4, *P* = 0.25, one-way ANOVA. Fraction venous endothelial cells across brain regions: *F*(8,18) = 1.2, *P* = 0.34, one-way ANOVA. Fraction tip-like endothelial cells across brain regions: *F*(8,18) = 1.4, *P* = 0.28, one-way ANOVA. **E** Differential expression analysis comparing capillary endothelial cells across brain regions, with region abbreviations as defined in (**A**). Volcano plots show genes with region-enriched and region-depleted expression in the region of interest compared to all other regions. Points are shown for all genes with average expression >1000 pseudobulk counts; colored points indicate genes with statistically significant enrichment or depletion (average expression >1000 psuedobulk counts, *P* < 0.05 DESeq2 Wald test (two-sided) with Benjamini-Hochberg correction). **F** UMAP plot of all endothelial cells, with points colored based on expression of *Stra6*. Color bar indicates expression (log-normalized counts). **G** Expression (log-normalized counts) of *Stra6* in striatal capillary, arterial, venous, and tip-like endothelial cells. **H** Expression (log-normalized counts) of *Stra6* and hippocampal-enriched receptor mediated transcytosis targets (*Lrp8*, *Igf1r*) in capillary endothelial cells across brain regions. Points represent average expression in each biological replicate and error bars represent SD. Source data are provided as a Source Data file. Gene expression data are provided in Supplementary Data 2.

We next aimed to determine whether there are regional specializations of the BBB in smaller, functionally distinct brain regions. We performed scRNA-seq on ECs acutely purified from nine dissected brain regions (cortex, hippocampus, Cb, SC, striatum, thalamus, hypothalamus, midbrain, and medulla/pons) to identify both the inter- and intra-regional heterogeneity. This experiment was repeated by two independent experimenter teams, with a total of three biological replicates collected for each brain region. Cluster analysis on the combined dataset revealed nine clusters (Supplementary Fig. 2A–E), which correlated with previously identified arterial/capillary/venous zonation^3^. Each brain region was represented in each cluster, demonstrating that the primary heterogeneity is intra-regional and dependent on location within the vascular tree (Fig. 1A–C, Supplementary Fig. 2). We did not observe differences in the relative abundance of unbiased clusters (Supplementary Fig. 2D) or collapsed arterial, capillary, venous, and tip-like endothelial subtypes across the 9 profiled regions (Fig. 1D).

We next evaluated inter-regional heterogeneity by comparing the gene expression of the capillary EC clusters. In a transcriptome-wide comparison, SC, medulla/pons, and midbrain formed one top-level hierarchical cluster distinct from other regions (Supplementary Fig. 3A). Furthermore, we identified genes that were enriched in capillary ECs in each region compared to all other capillary ECs (Fig. 1E, Supplementary Fig. 3B, Supplementary Data 2), as well as genes that were deficient in capillary ECs in specific regions (Fig. 1E, Supplementary Fig. 3C, Supplementary Data 2). We also identified 415 genes that showed significant differences in capillary ECs between regions in at least one pairwise comparison, with the hippocampus and striatum appearing most distinct from other regions (Supplementary Fig. 4, Supplementary Data 3). Therefore, there is indeed significant regional heterogeneity of CNS endothelial cell gene expression. In Fig. 1E, we present the most robust differentially expressed CNS endothelial genes. We found no significant differences in genes encoding many tight junction proteins (*Cldn5*, *Cldn12*, *Ocln*, *Lsr*, *Marveld2*) suggesting that the paracellular barrier was similar in each of the different brain regions. We did find regional differences in molecular transporters (*Slc16a1* enriched in cortex and hippocampus and depleted in medulla/pons and SC; *Stra6* enriched in striatum and depleted in midbrain; *Abca1* enriched in striatum), extracellular matrix (*Fn1* enriched in the hippocampus; *Sparc* enriched in the SC), immune modulators (*Bst2* and *Ifit3* enriched in midbrain; *Ifitm3* enriched in midbrain, medulla/pons and SC and depleted in cortex and hippocampus), transcytosis modulators (*Cav1* enriched in the SC and depleted in hippocampus), transcriptional regulators (*Foxq1* enriched in the hippocampus; *Tbx1* enriched in the striatum, hypothalamus, and SC and depleted in midbrain and Cb; *Ets1* enriched in the hippocampus), histone modulators (*Gse1* enriched in hippocampus), and signalling components (*Cpe* enriched in the Cb; *Piezo1* enriched in the thalamus) (Fig. 1E–H, Supplementary Fig. 3, Supplementary Data 2). Although there were fewer regionally-enriched/depleted genes in arterial and venous ECs than in capillary ECs, several genes exhibited enrichment/depletion in multiple branches of the vascular tree (*Tbx1* depleted in Cb capillary and arterial ECs; *Cpe* enriched in Cb capillary and venous ECs; *Rbp1* depleted in cortex capillary and venous ECs) (Supplementary Data 2).

Interestingly, hippocampal capillary ECs had many enriched genes involved in receptor-mediated transcytosis including *Tfrc, Igf1r*, and *Lrp8* (Fig. 1E, H, Supplementary Fig. 3, Supplementary Data 2). This is very interesting as several of these receptors are used as Trojan horse targets to receptors to aid in drug delivery to the CNS. To assess the functional consequences of the observed regional differential expression of *Tfrc*, we measured the uptake of a radiolabeled anti-transferrin receptor antibody. This antibody displayed preferential uptake in the hippocampus, cortex, and striatum compared to thalamus and Cb, similar to the scRNA-seq data (Supplementary Fig. 5). These data suggest that specific serum components, such as transferrin-iron, insulin and lipoproteins, may have differential access to different regions of the CNS, and that Trojan Horse drug delivery strategies utilizing these receptors may have differential efficacy in different brain regions with the greatest efficacy within the hippocampus. Bioinformatic analysis revealed pathways enriched/depleted in the capillary ECs of each region including enrichment of T cell receptor signaling pathway in the SC, depletion of chemokine signaling pathway in hippocampus, and enrichment of hedgehog signaling in the medulla/pons (Supplementary Data 2). Taken together, these data have identified that there is indeed significant inter-regional heterogeneity of EC gene expression among different brain regions and suggest that this heterogeneity may allow the BBB to differentially regulate the composition of each brain region through specializations in transport, metabolism, extracellular matrix and molecular signalling.

### Regulation of Stra6 expression at the BBB

**Fig. 2:**
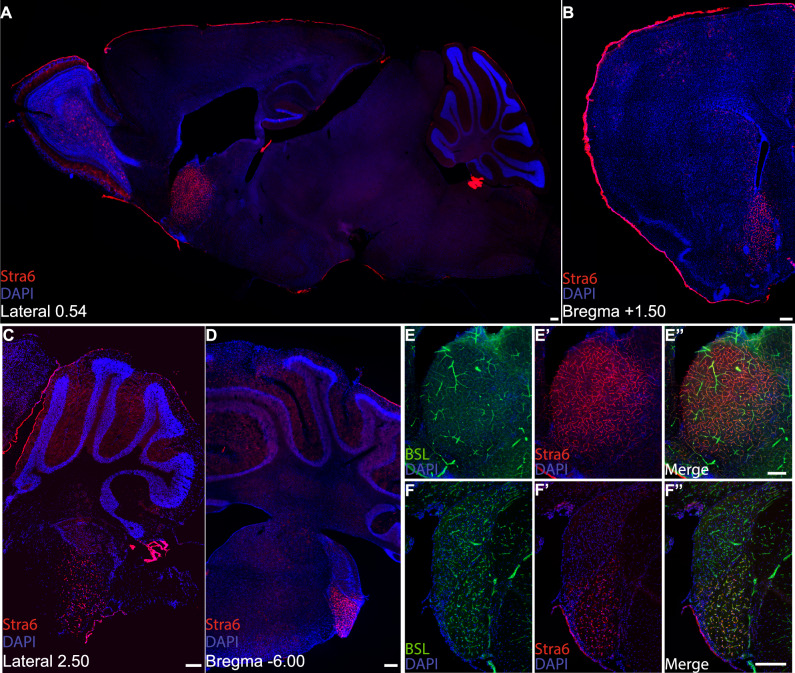
Stra6 expression within the CNS. **A–D** Sagittal (**A**, **C**) and coronal (**B**, **D**) sections of adult mouse brains were stained with an antibody directed against Stra6 (red) and DAPI to stain nuclei (blue). **E**, **F** Tissue sections of the rmShNAc (**E**) and PVCN (**F**) were stained with an antibody against Stra6 (red), with BSLI-Fluorescein (green) to label endothelial cells, and DAPI to label nuclei. Stra6 displayed vascular staining in the rmShNAc (**A**, **B**) and in the PVCN (**C**, **D**). All scale bars: 200 µm. Similar results were obtained in five independent experiments.

We next aimed to determine whether regional specifications of the BBB may regulate the local environment of the specific brain region. We analysed the expression of one striatum-enriched transcript: stimulated By Retinoic Acid 6 (*Stra6*). Stra6 is a 9-pass transmembrane protein that regulates ROL transport across cellular membranes^11–13^. ROL is the circulating form of vitamin A that is stored as retinyl esters in the liver and is secreted into the blood bound to ROL binding protein (RBP). Cell surface Stra6 binds to RBP-ROL complexes, leading to the dissociation of the ROL, whereby Stra6 further facilitates the transport of ROL across cell membranes^11–15^. Using immunohistochemistry, we found that Stra6 expression in CNS ECs in adult mice is robustly expressed in two distinct regions: the rostral part of the nucleus accumbens medial shell (rmShNAc) within the striatum and the posterior part of the ventral cochlear nucleus (PVCN), with sparse weak expression in other brain regions (Fig. 2A–D and Supplementary Fig. 6). In addition, Stra6 is expressed in specific non-vascular cells including meningeal fibroblasts, choroid plexus epithelial cells, and retinal pigment epithelial cells (Supplementary Figs. 7 and 8). This expression was consistent in multiple mouse strains including C57BL/6, Swiss Webster, and BALB/c (Supplementary Fig. 9).

**Fig. 3:**
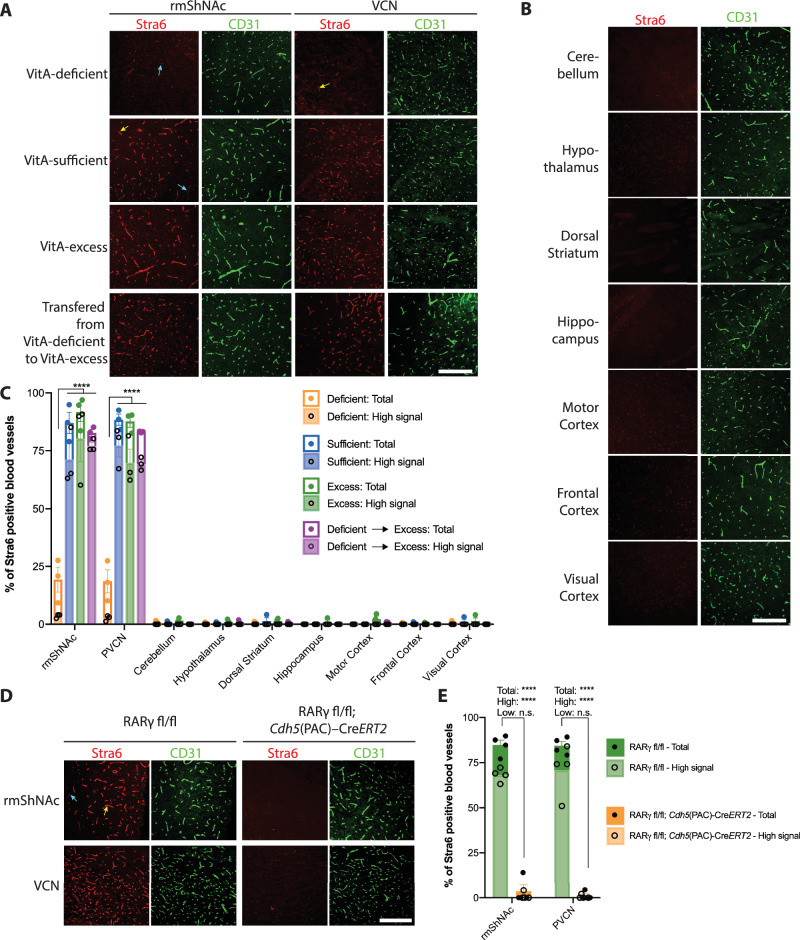
Stra6 expression at the blood-brain barrier is dynamically regulated by dietary amount of Vitamin A through RARƔ. **A** Tissue sections of the rmShNAc and VCN from 6 week old C57BL/6 mice raised on either VitA-deficient diet, VitA-sufficient diet, VitA-excess diet or raised on VitA-deficient diet then transferred to VitA-excess diet for 1 week prior to analysis, were stained with antibodies against Stra6 (red) and CD31 (green). Yellow arrows indicate vessels with high signal, blue arrows indicate vessels with low signal. Scale bar: 200 µm. **B** Tissue sections of the different brain regions from C57BL/6 mice raised on VitA-excess diet stained against Stra6 (red) and CD31 (green). Scale bar: 200 µm. **C** Percentage of vascular length positive for Stra6 in different brain regions according to different diet paradigm: VitA-deficient diet, VitA-sufficient diet, VitA-excess diet and VitA-deficient diet transferred to VitA-excess diet for 1 week. (*n* = 3 mice per region and diet). The vascular length with both high signal and low signal was quantified for each brain region. Statistics and p-values: two-way ANOVA (region *P* < 0.0001, diet *P* < 0.0001, interaction *P* < 0.0001) followed by a Tukey’s multiple comparisons test: rmShNAc VitA-deficient versus VitA-sufficient, VitA-deficient versus VitA-excess, and VitA-deficient versus VitA-deficient transferred to VitA-excess, total signal ****P < 0.0001; PVCN VitA-deficient versus VitA-sufficient, VitA-deficient versus VitA-excess, and VitA-deficient versus VitA-deficient transferred to VitA-excess, total signal ****P < 0.0001. **D** Tissue sections of the rmShNAc and PVCN from 6 week old RARg fl/fl; *Cdh5*(PAC)-CreERT2 and RARg fl/fl littermate controls that were raised on VitA-deficient diet, injected with tamoxifen at 4 weeks of age, and switched to a VitA-excess diet at 5 weeks, were stained with antibodies against Stra6 (red) and CD31 (green). Scale bar: 200 µm. Yellow arrows indicate vessels with high signal, blue arrows indicate vessels with low signal. **E** Percentage of vascular length positive for Stra6 in rmShNAc and PVCN in RARg ECK (RARg fl/fl; *Cdh5*(PAC)-CreERT2, *n* = 6) mice and in their littermate controls (RARg fl/fl, *n* = 7). The vascular length with both high signal and low signal was quantified for each brain region. Statistics and p-values: two-way ANOVA (region *P* = 0.77, genotype *P* < 0.0001, interaction *P* = 0.88) followed by Tukey’s multiple comparisons test: rmShNAc RARg fl/fl versus RARg fl/fl; *Cdh5*(PAC)-CreERT2, total signal ****P < 0.0001, high signal ****P < 0.0001, low signal *P* = 0.097; PVCN RARg fl/fl versus RARg fl/fl; *Cdh5*(PAC)-CreERT2, total signal ****P < 0.0001, high signal ****P < 0.0001, low signal *P* = 0.17. Error bars represent SEM. Source data are provided as a Source Data file.

Retinoids (vitamin A metabolites) can induce the expression of Stra6 itself^16,17^, therefore we examined EC Stra6 expression in mice raised on a vitamin A (VitA)-deficient diet (0 IU/kg), a VitA-sufficient diet (2400 IU/kg), and a VitA-excess diet (14,000 IU/Kg). We observed robust Stra6 expression in ECs of the rmShNAc and PVCN in both the VitA-sufficient and VitA-excess diet paradigms, however Stra6 expression was significantly decreased in ECs of these brain regions in mice raised on the VitA-deficient diet (Fig. 3A). Mice raised on a VitA-deficient diet, but switched to a VitA-excess diet for one week displayed EC Stra6 expression similar to that of mice raised on the VitA-excess diet, suggesting that VitA dynamically regulates the levels of Stra6 in the ECs of the rmShNAc and PVCN (Fig. 3C). Interestingly, dietary VitA did not affect Stra6 expression in the meninges or choroid plexus (Supplementary Fig. 8). When mice were raised on a conventional diet and then switched to the VitA-sufficient diet or VitA-excess diet at weaning, and examined at 10 weeks, there was little effect on robust EC-Stra6 expression in the rmShNac and PVCN. Interestingly, in this paradigm there was a decrease in the sparse EC Stra6 expression in some of the other brain regions tested with lower VitA (Supplementary Fig. 10), indicating that the sparse EC Stra6 in these brain regions may have a different threshold for maintenance by dietary VitA.

VitA/retinoic acid signalling is mediated by a series of nuclear receptors, primarily RARs (RARα/β/γ) that heterodimerize with RXRs (RXRα/β/γ) to regulate transcription^18^. Previously we have found that RARα and RARγ are expressed by CNS ECs^19^, and our combined scRNA-seq dataset indicates that *Rara* and *Rarg* are expressed in CNS ECs throughout the vascular tree, with *Rara* showing enriched expression in the thalamus, and *Rarg* displaying enriched expression in the striatum and depleted expression in the thalamus (Supplementary Fig. 11). To determine which nuclear receptor may regulate the VitA-dependent expression of EC Stra6, we examined whether there was a loss of EC Stra6 expression in RARα-null, RARβ-null, and tamoxifen-sensitive EC-specific RARγ conditional knockout mice. For these initial experiments all mice were raised on the conventional diet, examined at 10 weeks and compared to littermate controls. EC-specific RARγ conditional knockout mice and littermate controls were injected with tamoxifen at weaning. We found that there was no decrease in CNS EC Stra6 expression in RARα-null or RARβ-null mice fed conventional diet, however, there was a moderate, but incomplete, loss of EC Stra6 expression in the rmShNAc and PVCN of the EC-specific RARγ conditional knockout mice (Supplementary Fig. 12). To determine if the incomplete loss was due to partial redundancy of other nuclear receptors or residual Stra6 following tamoxifen-induced excision of RARγ, we developed a dietary paradigm to conditionally delete RARγ from ECs prior to induction of EC Stra6 by dietary VitA. EC-specific RARγ conditional knockout mice and littermate control mice were raised on a VitA-deficient diet starting at E14, tamoxifen was injected at 4 weeks, mice were switched to VitA-excess diet at 5 weeks, and analysed at 6 weeks age. In this paradigm we observed an almost complete reduction in Stra6 expression in the ECs of the rmShNAc and PVCN in EC-specific RARγ conditional knockout mice versus littermate controls (Fig. 3D, E). Taken together, these data demonstrate that Stra6 expression at the BBB of the rmShNAc and PVCN is dynamically regulated by dietary VitA signalling mediated by RARγ.

### Stra6 regulates retinoid transport and ShNAc function

**Fig. 4:**
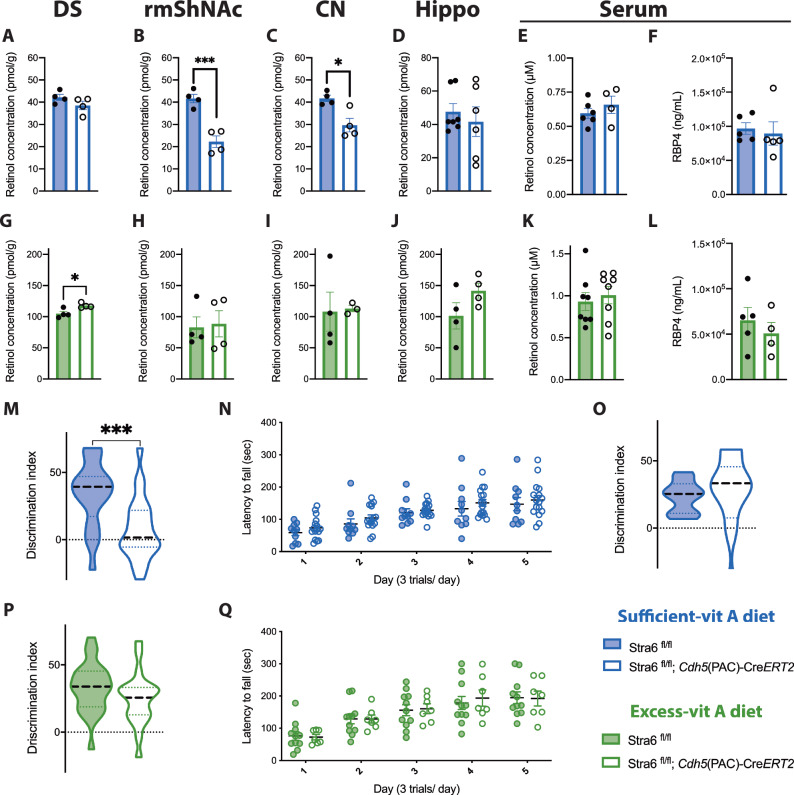
Endothelial specific Stra6 mutant mice display spatial memory defect due to a reduction of retinol uptake. **A**–**L** Retinols and RBP4 measurements from dissected brain regions or serum from endothelial-specific Stra6 knockout mice (Stra6 f/f; *Cdh5*(PAC)-*ERT2* and littermate controls (Stra6 f/f) raised on a VitA-sufficient diet (blue) or VitA-excess diet (green). Biological replicates are shown as individual data points. **A–C**, **G–J** *n* = 4 per genotype, **D** Stra6 f/f n = 7, Stra6 f/f; *Cdh5*(PAC)-*ERT2* *n* = 6, **E** Stra6 f/f *n* = 6, Stra6 f/f; *Cdh5*(PAC)-*ERT2* *n* = 4, **F** Stra6 f/f *n* = 5, Stra6 f/f; *Cdh5*(PAC)-*ERT2* *n* = 5, **K** Stra6 f/f *n* = 8, Stra6 f/f; *Cdh5*(PAC)-*ERT2* *n* = 8, **L** Stra6 f/f *n* = 5, Stra6 f/f; *Cdh5*(PAC)-*ERT2* *n* = 4. Statistics and p-values (**A–L**): unpaired two-tailed t-tests: **A** *P* = 0.17, **B** ***P = 0.0010, **C** *P = 0.012, **D** *P* = 0.55, **E** *P* = 0.38, **F** *P* = 0.72, **G** *P = 0.020, **H** *P* = 0.84, **I** *P* = 0.90, **J** *P* = 0.15, **K** *P* = 0.61, **L** *P* = 0.47. **M** Discrimination index for the novel location recognition task in endothelial-specific Stra6 knockout mice (Stra6 f/f; *Cdh5*(PAC)-*ERT2*, *n* = 23) and littermate controls (Stra6 f/f, *n* = 20) raised on a VitA-sufficient diet (blue). **N** Latency to fall in endothelial-specific Stra6 knockout (n = 16) and littermate control mice (*n* = 10) raised on VitA-sufficient diet (blue). **O** Discrimination index for the novel object recognition task in in endothelial-specific Stra6 knockout mice (*n* = 9) and littermate controls (*n* = 9) fed on VitA-sufficient diet. **P** Discrimination index for the novel location recognition task in endothelial-specific Stra6 knockout mice (*n* = 21) and littermate controls (*n* = 24) raised on a VitA-excess diet (green). **Q** Latency to fall in endothelial-specific Stra6 knockout (*n* = 7) and littermate control mice (*n* = 11) raised on VitA-excess diet (green). Statistics and p-values (**M–Q**): unpaired two-tailed t-tests: **M** ***P = 0.0008, **N** Day 1 *P* = 0.28, Day 2 *P* = 0.27, Day 3 *P* = 0.54, Day 4 *P* = 0.42, Day 5 *P* = 0.58, **O** *P* = 0.96, **P** *P* = 0.15, **Q** Day 1 *P* = 0.84, Day 2 *P* = 0.99, Day 3 *P* = 0.86, Day 4 *P* = 0.64, Day 5 *P* = 0.94. **A–L**, **N**, **Q** Error bars represent SEM. **M**, **O**, **P** Dashed lines indicate median and dotted lines indicate first and third quartiles. Source data are provided as a Source Data file.

We next aimed to test the hypothesis that Stra6 delivers retinoids specifically to the rmShNAc and PVCN. We generated EC-specific Stra6 knockout mice using *Cdh5*(*PAC*)–*CreERT2* and Stra6 floxed strains, and validated that this model deletes Stra6 from ECs but not other cell types in which *Stra6* is normally expressed (meningeal fibroblasts, choroid plexus epithelium, retinal pigment epithelium, olfactory neural cells) (Supplementary Fig. 7 and 8). EC-specific deletion of Stra6 did not increase BBB permeability to the small molecular tracer Sulfo-NHS-Biotin (Supplementary Fig. 13), demonstrating that Stra6 is not required for paracellular barrier properties of CNS ECs. EC-specific deletion of Stra6 led to a significant decrease in retinoids in the rmShNAc and cochlear nucleus (CN) of mice raised on sufficient VitA diet, but not in other brain regions including the dorsal striatum (DS) and hippocampus, or in the serum (Fig. 4A–E). These deficits were not observed when mice were fed a VitA-excess diet (Fig. 4G–L). Therefore, the specific localization of Stra6 at the BBB of the rmShNAc and PVCN acts to transport retinoids to these brain regions, however feeding excess VitA can largely overcome the requirements of EC Stra6.

We next aimed to determine whether Stra6-mediated concentration of retinoids in the rmShNAc is required for local brain function. The nucleus accumbens (NAc) is a part of the basal ganglia located in the ventral striatum. It is critical for motivation, aversion and reward reinforcement and plays an important role in addiction^20–23^. The NAc can be divided into the core (cNAc) and the shell (ShNAc). The cNAc regulates slow wave sleep and reward relating to motor function, whereas the ShNAc regulates the cognitive processing of reward, including preference for pleasurable stimuli and reinforcement learning^20,24–26^. The ShNAc has also been shown to regulate spatial learning, as lesioning the ShNAc or injections of either D1 or D2-type receptor antagonists into the NAc leads to deficits in spatial learning tasks^27–30^. We therefore investigated the impact of *Stra6* EC-specific conditional deletion in three behavioural paradigms: i) spatial memory using a novel location recognition task which displays deficits when the ShNAc is lesioned^30^, ii) the novel object recognition task, which displays deficits when the cNAc is lesioned^30^ and iii) motor learning using the accelerating rotarod which is mediated by the neighbouring DS.

EC-specific deletion of Stra6 resulted in a significant deficit in the novel location recognition task in mice fed a VitA-sufficient diet. Control mice fed the VitA-sufficient diet explored with a discrimination index close to 50, indicating a strong preference for the displaced object, whereas EC-specific Stra6 mutants fed the VitA-sufficient diet explored at a discrimination index close to 0, indicating no preference for the displaced object (Fig. 4M; Supplementary Fig. 14). We did not observe a deficit in novel object recognition or accelerating rotarod tasks in the EC-specific Stra6 knockout mice, suggesting that the phenotype is specific to spatial memory (Fig. 4N, O, Q). The spatial learning phenotype was rescued when mice were fed the VitA-excess diet, demonstrating that EC expression of Stra6 regulates spatial memory through VitA/ROL signalling (Fig. 4P; Supplementary Fig. 14). These data suggest that Stra6 plays an important role in regulating the region-specific levels of retinoids, which may be most critical when individuals have low levels of VitA in their diet. Indeed, human mutations in Stra6 lead to devastating phenotypes including anophthalmia, congenital heart defects, cognitive dysfunction and other systemic issues^31^. The ability of increased dietary VitA to abrogate the need of Stra6 is consistent with the presence of Stra6-independent methods for VitA/retinoids to enter tissues, perhaps either in chylomicrons or following passive diffusion if free retinoids can dissolve in the aqueous serum at high concentrations^11–13^.

## Discussion

In this study, we used a combination of EC purification and scRNA-seq to profile ECs from nine different regions of the CNS, allowing us to understand both inter-regional heterogeneity and intra-regional heterogeneity. Unbiased clustering revealed that the cells did not cluster by brain region, as each of the brain regions was represented in each of the clusters. Instead, the unbiased clusters represent different levels of the vascular tree from arterioles to capillaries to venules, which have been identified in previous single cell sequencing studies on the vasculature^3^. This demonstrates that intra-regional heterogeneity is greater than inter-regional heterogeneity, as the largest determinants of endothelial gene expression is based on where the EC is in the vascular tree, and not where the EC is within the CNS. This is not surprising as it is clear that there are very important structural and functional differences between ECs at different levels in the vascular tree, with arterioles critical regulators of vascular tone and blood flow, capillaries the most important mediators of exchange between the blood and the underlying tissues, and post-capillary venules being the key mediators of immune cell interactions^3–9^. Further, differences in cell shape, curvature, and association with mural and immune cells are observed between each of the different levels of the vasculature tree.

While it is known that specific regions of the CNS, termed the circumventricular organs, lack a BBB and instead have fenestrated vessels that permit the robust exchange of solutes between the blood and these neurosensory and neurosecretory organs^10,32–34^, very little is known about whether there is regional heterogeneity between ECs in regions of the CNS that have a functional BBB. Despite the fact that we observed that intra-regional heterogeneity was greater than inter-regional heterogeneity ECs, we still observed robust differences in endothelial gene expression between different regions of the CNS. This suggests that the BBB may in fact have regional specializations that may modulate the local environment to regulate the function of specific circuits. Interestingly, it has been shown that different components of the Wnt/β-catenin signalling pathway, including ligands, frizzled receptors, and co-receptors, are required for induction and maintenance of the BBB in different parts of the CNS. For instance, the ligand Norrin drives the formation of the blood-retinal barrier, Wnt7a/7b are required for BBB formation in the ventral brain and SC, and Wnt1/3/3a/4 have been suggested as candidates for BBB induction in the dorsal CNS^35–40^. However, it is not clear whether different Wnt/β-catenin signalling components lead to differences in endothelial gene expression and BBB function, or are remnants of developmental dorsal-ventral and rostral-caudal patterning.

Regional heterogeneity of blood vessels may reflect the differential requirement of each region for oxygen, nutrients, and molecular signals, due to the heterogeneity of cell populations and function of each region. For instance, there are clear differences in vascular density between the gray and white matter^41,42^, with gray matter having much denser vascular networks due to the elevated energy requirements of synaptic circuitry compared to myelinated axons. Interestingly, our data did not show obvious clustering of ECs from regions with large amounts of white matter (SC, Cb) compared to those with lower amounts of white matter (cortex, hippocampus, hypothalamus), indicating that white matter versus gray mater may not be the key determinant of the regional heterogeneity of the endothelial gene expression. The hippocampus is a brain region with distinct vascular cell interactions, as this region has stem cells that persist into adulthood that reside in a vascular niche, with the ECs being key modulators of neural stem cell self-division and differentiation. Interestingly, we found robust enrichment of genes within ECs of the hippocampus compared to other regions, including *Tfrc*, *Igf1r*, *Lrp8*, *Ctla2*, and many other genes. It will be interesting to identify whether enrichment of these genes is required to mediate endothelial-stem cell interactions in the hippocampus, and whether these same genes are enriched in the subventricular zone, another region with ongoing neurogenesis in adults.

Our data further demonstrate robust enrichment of *Stra6*, a retinoid transport regulator, in the BBB of the rmShNAc and the PVCN, and that conditional knockout of *Stra6* from the endothelium leads to a decrease in retinoids in these brain regions, but not the blood or other brain regions. These data confirm that indeed regional specializations of the BBB may be important regulators of the local neural environment. Interestingly, we found that feeding mice a Vitamin A-excess diet could rescue the levels of retinoids in these brain regions, suggesting that there are additional methods for delivery of retinoids that become redundant when systemic retinoid levels are elevated. This could indicate the presence of alternative retinoid transporters or passive diffusion of free lipophilic retinoids. What remains unclear is why the rmShNAc and the PVCN would require this specific mode of retinoid transport. Retinoids modulate many different biological processes including cell division, cell differentiation, cell motility and cell–cell signaling^43–45^. In particular, retinoids have been shown to regulate homeostatic synaptic plasticity in different circuits within the brain^46,47^, however it is not clear whether there is enrichment of Stra6 in the rmShNAc and PVCN because these brain regions have unique homeostatic synaptic plasticity function.

We demonstrated that dietary Vitamin A modulated the expression of Stra6 at the BBB of the rmShNAc and the PVCN, and this was mediated through RARƔ signaling within the ECs. This suggests a feedforward mechanism, whereby systemic retinoids signal to the ECs in these brain regions to increase Stra6 expression, allowing more retinoids to enter these brain regions. While it is clear that dietary Vitamin A is a key modulator of Stra6 expression at the BBB, this cannot dictate the regional heterogeneity of Stra6 expression as the lumen of the blood vessels in each of the brain regions would encounter similar systemic factors. Therefore, there are likely local cues derived from within the rmShNAc and the PVCN that make them specifically poised to respond to systemic retinoids. Indeed, we found enrichment of RARƔ expression within the striatum. Combined with the finding that conditional knockout of RARƔ within the endothelium greatly reduces Stra6 expression, this suggests regional heterogeneity of RARƔ expression as a potential mechanism by which the endothelium is poised to respond to systemic retinoids; however, the mechanism that drives regional RARƔ expression is still unknown. Taken together, our data indicate that regional specializations of the BBB may be sculpted by a combination of local and systemic cues including diet. Interestingly, it is becoming more clear that peripheral factors may influence BBB function, including diet and exercise. For instance, dietary induced ketogenesis increases monocarboxylic acid transporters at the BBB^48^. Furthermore, systemic Netrin signaling through endothelial Unc5b is thought to modulate Wnt/β-catenin induction of BBB properties^49^. Moreover, neural activity is found to be a key driver of BBB gene expression, including modulating the expression of efflux transport and Igf1 transport^50,51^. Therefore, the BBB can be thought of as a dynamic interface between the blood and neural tissue, that mediates the exchange between the two systems in response to signals from both sides.

We also identified a large number of transcription factors, signalling molecules, and metabolic enzymes are also regionally enriched, highlighting the different mechanisms exploited by the BBB to regulate the composition of the surrounding neural tissue. Studying these BBB specializations may give important insight into the mechanisms that modulate the function of specific brain regions, and may also provide molecular ‘‘sign-posts’’ to guide localized drug delivery. Furthermore, many current approaches for CNS drug delivery platforms utilize antibodies that bind to the luminal domains of BBB-specific transmembrane proteins, such as the transferrin receptor, as ‘‘Trojan Horses’’ to take advantage of the receptor-mediated transcytosis to cross the BBB^52^. Indeed, we observed preferential uptake of an anti-transferrin receptor antibody in the hippocampus, striatum, and cortex compared to hypothalamus and Cb. Identification of region-specific transmembrane proteins may help guide brain region-specific drug delivery, by acting as sign-posts to either guide Trojan Horses to specific brain regions, or as the receptor for the Trojan Horses themselves. For instance, Stra6 may provide an extracellular epitope to enrich targeting therapeutics to the rmShNAc and PVCN compared to other brain regions.

## Methods

### Animals

Animal experiments were conducted in accordance with protocols approved by the University of California, San Diego Institutional Animal Care and Use Committee (protocol number S14044) or Université Laval Animal Use Committee (protocol number CPAUL, 20-625). Mice were housed in a 12:12 light/dark cycle with ad libitum access to water and food at ~21 °C and ambient relative humidity. Unless noted animals were feed on PicoLab Rodent Diet 20 (Lab Diet, Vitamin A content: 15,000 IU/kg). Tie2GFP mice (Jax stock# 003658), Cg-Gt(ROSA)26Sor<tm9(CAG-tdTomato)Hze mice (Jax stock# 007909), RARalpha knockout (Jax stock# 023845) and RARbeta knockout (Jax stock# 022999) mice were acquired from Jackson labs. Stra6 floxed mice (MGI: 5532504) and RARgamma floxed mice (MGI:2386111) were generously provided by Norbert B. Ghyselinck. Tg(Cdh5-cre/ERT2) (MGI:3848982) mice were kindly provided by Ralf H. Adams.

### Analysis of dietary regulation of Stra6 expression

In order to assess the impact of dietary VitA uptake on Stra6 expression at the BBB, C57BL/6 wild-type mice were raised and bred on conventional rodent chow. At E14 dams were transferred to a either a VitA-deficient diet (VitA 0 IU/kg, AIN-93G, TD.10991, Teklad Envigo) a VitA-sufficient diet (VitA 2400 IU/kg, AIN-93G, D.170361, Teklad Envigo) or a VitA-excess diet (VitA 14,000 IU/kg, AIN-93G, TD.180256, Teklad Envigo), and weaned mice were raised on the same diet until analysis at 6 weeks of age. To assess whether Stra6 is dynamically regulated by VitA, mice were raised on a VitA-deficient diet and transferred to a VitA-excess diet at 5 weeks of age, prior to analysis at 6 weeks old. In order to identify which RAR nuclear receptors regulate Stra6 expression at the BBB, RARalpha heterozygous breeding pairs, RARbeta heterozygous breeding pairs and RARgamma fl/fl; Cdh5-cre/ERT2/+ mice crossed with RARgamma fl/fl breeding pairs were housed with conventional rodent chow (PicoLab Rodent Diet 20, containing 15,000 IU/kg of VitA), and their offspring were weaned on the same diet and analysed at 8 weeks of age. In addition, RARgamma fl/fl; Cdh5-cre/ERT2/ + mice crossed with RARgamma fl/fl were mated on conventional rodent chow and at E14 dams were transferred to a VitA-deficient diet, and pups were weaned on a VitA-deficient diet. EC-specific RARg knockout mice (RARgamma fl/fl; Cdh5(PAC)-CreERT2/+) and littermate controls (RARgamma fl/fl) were given an IP injection of tamoxifen (100 µL, 20 mg/mL) for 3 consecutive days at 4 weeks of age, transferred to the VitA-excess diet at 5 weeks of age and analyzed at 6 weeks of age. For behavioral experiments, endogenous ROL measurements, serum RBP4 measurements, BBB permeability assay and validation of the Stra6 endothelial specific KO mice, breeding pairs of Stra6 endothelial specific inducible conditional knockout mice were kept on conventional rodent chow and pups were transfered to either a VitA-sufficient diet or a VitA-excess diet at weaning. Tamoxifen was administered through an IP injection (100 µL, 20 mg/mL) for 3 consecutive days at weaning for endothelial specific deletion of Stra6.

### Immunofluorescence

Mice were put under general anaesthesia using a Ketamine/Xylazine mixture and perfused with Phosphate Buffered Saline (PBS) for 2 min, followed by a 5 min paraformaldehyde 4%-PBS perfusion. Tissue were collected, cryoprotected in 30% sucrose-PBS overnight at 4 °C and frozen in a mixture OCT and 30% sucrose (2:1 ratio). Cryosections were blocked with 50% serum, permeabilized with 0.5% Triton X-100, and stained with adequate concentration of primary (Rabbit anti-Stra6, 1:1000, Abgent cat# AP9433b; Rat anti-CD31, 1:1000, BD cat# 553370) and secondary antibodies (Goat anti-Rabbit IgG and Goat anti-Rat IgG Cross-Adsorbed, Life technologies). Blood vessels stained with Fluorescein Griffonia (Bandeirea) Simplicifolia Lectin 1 (1:1000, Vectorlabs). Slides were mounted in DAPI-Fluoromount-G and visualized with a digital camera (Axiocam HRm; Carl Zeiss) connected to an epifluorescence microscope (Axio Imager D2; Carl Zeiss). The acquisition software used was AxioVision (Carl Zeiss) and images were processed using Photoshop CS6 (Adobe). The percentage of Stra6 positive blood vessels was quantified using Image J using a Threshold function in ImageJ to set upper and lower thresholds to identify vessels with low and high signal. Using the “freehand tracing” tool and the “ROI Manager”, Stra6 positive blood vessels of high signal intensity were traced and validated with the corresponding CD31 positive image. Once completed the threshold was removed and Stra6 positive blood vessel of low signal intensity were traced. To identify the Stra6 negative blood vessels, the traced blood vessels were overlayed to a CD31 channel image and the remaining vessels were traced. After each step, the sum of length of traced blood vessels was computed with Measure function in ROI Manager.

### BBB permeability assay

Anesthetized mice underwent a 10 min intra-cardiac perfusion of NHS-biotin (50 mg/250 mL DPBS) followed by 10 min of 4% PFA perfusion. Brain were harvested and cryoprotected in 30% sucrose overnight at 4 °C and frozen in a mixture OCT and 30% sucrose (2:1 ratio). Biotin was detected by streptavidin-Alexa 488 and fluorescence signal intensity was measured by fluorescence microscopy and analysed using Fiji. Biotin leakage was assessed by measuring the mean fluorescence intensity within an area corresponding to the entire region of interest.

### ECs FACS purification

All mice used for bulk and scRNA-seq were male. For bulk RNA sequencing of brain, Cb, SC, liver and lung ECs, ECs were purified from homozygous Tie2GFP mice at 10–14 weeks of age^53,54^. For single-cell RNA sequencing, CNS ECs were purified from Cg-Gt(ROSA)26Sor<tm9(CAG-tdTomato)Hze; Tg(Cdh5-cre/ERT2) mice that were given IP injections of tamoxifen (100 µL, 20 mg/mL) for 3 consecutive days one week before endothelial cell purification at 10–14 weeks of age^19^. Briefly, mice were anesthetized with ketamine/xylazine, euthanized by decapitation, brain and SC harvested, meninges removed, and brain dissected in order to isolate different regions. The tissue was finely minced with a scalpel blade and digested in papain solution (10 mL of EBSS, supplemented with 0.46% D(+)-Glucose, 26 mM NaHCO_3_, 0.5 mM EDTA with 1 vial of papain (120U, Worthington-biochem, cat #LK003176)) for 1.5 h at 37 °C. Mechanical trituration was performed, then another enzymatic digestion with Collagenase II (1.0 mg/ml; Worthington-Biochem, cat #LS004174) and neutral Dispase (0.4 mg/ml; Worthington-biochem, cat # LS02100) in 10 mL EBSS, supplemented with 0.46% D(+)-Glucose, 26 mM NaHCO_3_, 0.5 mM EDTA was done to obtain a single cell suspension. MACS magnetic beads conjugated to anti-myelin antibody (Miltenyi cat #130-096-433) were used to remove oligodendrocytes and remaining myelin-wrapped axons/neurons. ECs were FACS isolated for tdTomato^+^ fluorescence and using CD13 (FITC Rat anti mCD13 clone R3-242, BD Pharmingen cat #558744) and DAPI for negative selection of pericytes and dying cells. For bulk RNA sequencing experiment, ECs were gated for GFP, collected in Trizol, and RNA extraction and purification was performed using RNeasy Micro Kit (Qiagen). For scRNA-seq, ECs were collected in 0.05% albumin PBS solution.

### Bulk RNA-seq of ECs

#### RNA isolation, library construction, and RNA sequencing

Total RNA was extracted from Cb, Fb), and SC, liver and lung ECs using TRIzol reagent (Invitrogen) following the manufacturer’s instructions. A Bioanalyzer (Agilent) was used to determine the quality of RNA samples. Poly-A selected RNA samples were used to construct 100 bp paired-end sequencing libraries using the TruSeq RNA Sample Prep Kit (Illumina). Two biological replicates were prepared for each CNS endothelial cell type. In order to compare ECs from the CNS with those from different tissues, we collected cells from liver and lung. Samples were sequenced using the Illumina HiSeq 2000 sequencer.

### Read mapping and quantification

The quality of all sequenced samples was addressed using FastQC. Read mapping, transcript assembly, and quantification were performed using a previously described pipeline^55^. Briefly, sequenced reads were mapped to the mm10 mouse reference genome using TopHat v2.1.0^56^ with default parameters. Aligned reads were assembled using Cufflinks v2.2.1^57^, and both annotated genes and transcripts were quantified as FPKM (Fragments Per Kilobase of transcript per Million mapped reads) values. We used a combined annotation file which was obtained by merging GENCODE M14 (www.gencodegenes.org) with NCBI lncRNA definitions (downloaded on 06/07/2017). A total of 21,948 protein-coding and 11,607 lncRNA genes were queried. To avoid inflation ratio, gene FPKM values < 0.1 were set to 0^58^. Genes with FPKM ≥ 1 in at least one of the replicates were considered “expressed.” Additionally, read counts for annotated genes and transcripts were obtained using HTSeq-count^59^. Similarity among replicates was calculated using the Pearson correlation of log2-transformed FPKM values of endothelial samples. A dendrogram was built using the Euclidean distances between the log2-transformed FPKM values of expressed and highly variable (standard deviation ≥ 2) genes from all endothelial cell samples. In the dendrogram, hierarchical clustering was performed implementing Ward’s linkage method.

### Differential gene expression analysis

Pairwise comparisons were performed (Fb vs Cb, Fb vs SC, Cb vs SC) using DESeq2^60^ with normalized counts to determine differentially expressed genes (DE). Venn diagrams were built to depict DE genes in each pairwise comparison. DE genes unique to an endothelial cell type were those common in the two pairwise comparisons involving the cell type (e.g upregulated in Fb vs Cb and upregulated in Fb vs SC). Genes were considered DE if they were expressed (FPKM ≥ 1 in at least one replicate) and their fold-change > 2 with p-value < 0.05. Only DE genes were considered for downstream analysis. A heatmap was generated with the log2-transformed normalized counts of DE genes.

### GO enrichment analysis

To understand the underlying biological functions of DE protein-coding genes, an enrichment analysis of GO terms was performed. Mouse GO terms were downloaded from http://bioinf.wehi.edu.au/software/MSigDB/ and gene set enrichment was calculated using a hypergeometric statistical test (phyper R function) with the list of DE upregulated genes for each endothelial cell type. Gene sets with *p* value < 0.00005 were considered enriched. Bar plots were used to depict the resulting gene sets including the number of genes and the enrichment *p* value.

### Single cell RNA-seq

#### EC isolation

Cb, cortex, hippocampus, hypothalamus, medulla/pons, SC, striatum and thalamus were dissected from mice homozygous for both Cg-Gt(ROSA)26Sor<tm9(CAG-tdTomato)Hze and Tg(Cdh5-cre/ERT2). Dissected regions from 3–6 mice were pooled to form each biological replicate. Two rounds of isolation were performed independently by different experimenters, with 1–2 biological replicates per round, for a total of three biological replicates per brain region (Supplementary Table 1). After the isolation of tdTomato^+^ ECs by FACS (Supplementary Fig. 15) the single cell suspension was immediately used for scRNA-seq. Cells from the first round of isolation (“Experiment A”) were prepared using the Single Cell Gene Expression V2 kit (10X Genomics) according to manufacturer instructions, and sequenced on an Illiumina HiSeq 4000. Cells from the second round of isolation (“Experiment B”) were prepared using the Single Cell Gene Expression V3.1 Kit (10X Genomics) and sequenced on an Illumina NovaSeq 6000.

### scRNA-seq analysis

The mouse reference genome was obtained from 10X Genomics (mm10-2020-A) and the *tdTomato* gene from the Ai9/Ai14 targeting vector^61^ (containing the tdTomato coding sequence, WPRE, and bGH poly(A) signal) was added to the reference using the CellRanger mkref function. FASTQ files were aligned to the reference genome and gene expression matrices generated using CellRanger (version 6.0.1). Gene expression matrices were imported into R (version 4.1.1), merged, and analyzed using the Seurat package (version 4.0.5.9003)^62,63^. Cells with fewer than 250 detected genes or greater than 5% of total counts derived from mitochondrial transcripts were removed. Variable feature identification was performed using the SCTransform package (version 0.3.2)^64^. The following parameters were included as variables to regress: batch (Supplementary Table 1), percentage of counts derived from mitochondrial transcripts, and immediate early gene score based on expression of *Fos, Fosb, Jun, Junb, Egr1, Egr2, Egr3, Ier2, Ier3, Dusp1, Atf3, Nr4a1*, and *Nr4a2*. Harmony^44^ was used to regress out batch effects. Graph-based clustering was used to identify and remove clusters of contaminating non-ECs, including pericytes/vascular smooth muscle cells, fibroblasts, immune cells, pericyte-EC doublets (*Cldn5*^+^*Rgs5*^high^), ECs containing erythrocyte mRNAs (*Cldn5*^+^*Hbb-bt*^+^), and non-BBB ECs (*Cldn5*^low^*Plvap*^+^). We also removed clusters that were likely artifacts of tissue preparation, based on overrepresentation in specific batches or enrichment for markers of low quality cells (e.g., heat shock protein genes *Hspa1a*, *Hspa1b*, *Hspb1*; ribosomal and/or mitochondrial genes). The resulting filtered dataset contained 44,427 CNS ECs.

We performed variable feature identification and Harmony regression on the filtered dataset and visualized cells using UMAP embedding. We performed unbiased graph-based clustering and identified differentially expressed genes in each cluster using the Wilcoxon rank sum test (Supplementary Fig. 2). We compared the average transcriptome profiles of these clusters based on Euclidean distances, calculated using the first 25 Harmony components. We annotated clusters as arterial, venous, and tip-like based on enrichment of multiple known markers of these endothelial subtypes (arterial: *Gkn3, Mgp, Clu, Vegfc, Stmn2*; venous: *Icam1, Vcam1, Lrg1, Slc38a5, Vwf*; tip-like: *Angpt2*, *Apln*, *Meox1*). We annotated remaining clusters as capillary. The resulting dataset contained 37,078 capillary, 3644 arterial, 3327 venous, and 378 tip-like ECs.

We performed further analyses on capillary ECs. We compared the average transcriptome profiles in each region based on Euclidean distances, calculated using the first 25 Harmony components. We identified genes enriched or depleted in each brain region compared to all other brain regions using a pseudobulk approach: For each biological replicate (Supplementary Table 1) we calculated the average expression of each gene in each region, using raw counts. We scaled all values by 10^4^ and rounded to obtain integer pseudobulk count values. We used DESeq2^39^ for differential expression analysis, comparing each region to all other regions. Experiment (A/B) was included in the DESeq2 design to regress out batch effects. Differentially expressed genes satisfied the criteria of *P*_adj_ < 0.05 (DESeq2 Wald test with Benjamini-Hochberg correction) and average expression across all cells > 1000 psuedobulk counts. Analogous differential expression analyses were performed for arterial and venous ECs. For capillary ECs, we also performed all pairwise comparisons between brain regions using the differential expression parameters described above. For visualization (bar plots), for each biological replicate we calculated the average expression of each gene in each region, using log-transformed counts. For Gene Set Enrichment Analysis (GSEA)^65^ we pre-ranked genes based on the log_2_(fold change) and used GSEA (version 4.1.0) to test ranked gene lists against the Hallmark^66^ and KEGG Pathways^67^ database. All code for scRNA-seq will be available upon request to authors.

### HPLC, LC-MS endogenous retinoids measurements

To measure ROL in the brain, each brain region was dissected, and then for the DS, rmShNAc, CN) (CN, containing the VCN) the brain regions from ten mice were first pooled together for each n; for the hippocampus and serum, each n represents one mouse. Animals were put under general anesthesia and perfused with ice-cold PBS for 4 min. Immediately after, blood was collected and brain dissected on ice-cold PBS under yellow light. Dissected brain samples were transferred to amber tubes and flash frozen on dry ice. Blood sat on ice for at least 30 min until centrifugation at 10,000 *g* for 10 min at 4 °C. Serum was collected, transferred to a new amber tube and flash frozen on dry ice. Samples were stored at −80 °C until endogenous retinoid extraction. The tissue or serum was then processed using a published liquid-liquid extraction protocol for mouse spleen ROL with minor modifications^68^. Briefly, pooled tissues were homogenized with 0.9% saline. For DS (50–80 mg), rmShNAc (15–35 mg) and CN (20–50 mg) regions, a 10 to 1 ratio of saline to tissue weight was used for homogenization. For HI and CE regions (130–180 mg), a 5 to 1 ratio of saline to tissue weight was used. Homogenates were transferred into glass tubes and added with 4 µL of 4 µM internal standard ROL-d_6_ (Cambridge Isotope Laboratories, Inc; Tewksbury, MA). After adding 2 mL of 0.25 mM KOH dissolved in EtOH and vortexing, 10 mL of hexanes was added for organic solvent extraction. The hexane layer was then transferred into another glass tube and dried under N_2_ flow, the dried residues were resuspended in 80 µL acetonitrile for LC-MS/MS analysis. To measure ROL in serum, an acetonitrile precipitation method was used. Mouse serum of 50 µL was added into Eppendorf tubes followed by the addition of 4 µL of 4 µM ROL-d_6_. Ice-cold acetonitrile of 100 µL was added followed by intense vortexing. Samples were left on ice for 10 minutes and then were centrifuged at 18,000 *g* at 4 °C for 30 min. The supernatant was used for LC-MS/MS analysis. The LC-MS/MS analysis was performed using an AB Sciex 5500 QTRAP mass spectrometer (AB Sciex LLC; Framingham, MA) coupled with an Agilent 1290 UHPLC (Agilent Technologies; Santa Clara, CA). The LC-MS/MS method for ROL measurement was the same as described previously^69^. A multiple reaction monitoring transition was added into the LC-MS/MS method for ROL-d_6_ (*m/z* 275 > 96). Detailed description and validation of retinoid measurement assays can found in previous studies^70^ and example chromatograms are shown in Supplementary Fig. 16.

### RBP4 measurements

RBP4 levels were measured using the Mouse RBP4 ELISA Kit from Abcam (cat # ab195459, Lot # GR3265123) according to the manufacturer instructions. Each *N* corresponds to the serum of a single mouse.

### Measurement of regional uptake of anti-transferrin receptor antibody

Transferrin receptor function was measured by in situ perfusion of a tritiated internalizing anti-transferrin receptor antibody (Ri7.217.1.4; ^3^H-Ri7). 3-month-old male C57BL/6 mice were perfused directly in the internal carotid artery with 0.60 µCi/ml (9 nM, 3.5 µg) of ^3^H-Ri7 for 1 min at a flow rate of 2.5 mL/min. The vascular marker ^14^C-sucrose was coperfused at 0.40 µCi/ml to subtract the vascular volume from Ri7 data and to evaluate BBB integrity. Immediately after the end of perfusion, mice were decapitated, and the right hemisphere was dissected (hypothalamus, hippocampus, striatum, Cb, cortex, and remaining white matter for the total hemisphere estimation) and weighed before overnight digestion in 1 mL Solvable. Liquid scintillation counting was performed with the perfusate and brain samples. Radioactive counts (^3^H dpm and ^14^C) in each brain region were used to calculate the volume of distribution (V_d_) and the uptake coefficient (CL_up_) of Ri7, and the vascular volume (^14^C-sucrose, V_vasc_).

### Behavioral paradigms

#### Novel object and novel location recognition memory task

Animals were handled for 2 min per day every day for 9 days to habituate them to the experimenter and to reduce stress and anxiety. The day before the experiment, the animals were habituated to the open field arena (50.8 × 50.8 × 38.1 cm^3^) for 60 s. On the day of the experiment, mice were placed in the empty open field arena for 5 min of habituation. The animals were then trained three times in the arena with two identical objects in set locations for 5 min each time, with a 3 min rest period between each training session. The test for the novel object recognition task is then performed by replacing one object by a new object, and then the mouse is placed back in the arena for 5 min. The novel location recognition test is performed by the displacement of one object to a new location in the arena and then the mouse is placed back in the arena for 5 min. All the movements of the mice were recorded on a camera during the 5 min test period and the recognition time for each object was measured by a blinded experimenter. Discrimination rate was calculated as [(time spent with the displaced/new object) - (time spent with the non-displaced/old object) / (time spent with the displaced/new object + time spent with the non-displaced/old object)]x100. Mice that didn’t reach 10 in total exploration time were excluded from the analysis.

### Rotarod

Each mouse was given three trials per day on the accelerating rotarod (Touchscreen Rota rod, Panlab) (4–40 rpm over 5 min) for 5 consecutive days, with a 90 s interval between trials. Before the trial was initiated, mice were trained the 4 rpm constant speed rotarod for 30 s. The latencies to fall were automatically recorded for each trial by a computer using Sedacom software (Panlab).

### Statistical analysis

Differential Stra6 expression, ROL levels, and behavioral data were statistically analyzed using GraphPad Prism v9 (GraphPad Software, La Jolla, CA, USA) and reported as mean ± standard error of the mean (SEM). Two-way ANOVA with a Tukey’s multiple comparison test was used to compare Stra6-positive vessel length in different brain regions of mice raised under different diet paradigms and between the Stra6-positive vessel length in endothelial specific RARƔ KO mice and their littermate controls. Unpaired two-tailed *t*-test (two groups) were used to analyze ROL levels, behaviors, the assessment of BBB permeability and the validation of endothelial specific Stra6 KO mice. *P ≤ 0.05, **P ≤ 0.01, ***P ≤ 0.001, ****P ≤ 0.0001, † *P ≤* 0.000001. Exact p-values are provided in figure legends except in cases when *P* < 0.0001 was reported by GraphPad Prism. Samples sizes were chosen based on similar experiments in the literature.

### Reporting summary

Further information on research design is available in the Nature Portfolio Reporting Summary linked to this article.

## Supplementary information

Supplementary Information

Description of Additional Supplementary Files

Supplementary Data 1

Supplementary Data 2

Supplementary Data 3

Reporting Summary

Transparent Peer Review file

## Source data

Source Data

## Data Availability

The bulk RNA-seq and scRNA-seq data generated in this study have been deposited in the Gene Expression Omnibus repository with accession codes GSE171105 and GSE165457, respectively. scRNA-seq data are additionally available in an interactive format at http://danemanlab.shinyapps.io/bbbtranscriptome. Source data are provided with this paper.
