# Supplementary material for: Regional heterogeneity of the blood-brain barrier
Source: Nat Commun. 2025 Aug 8;16:7332. doi: 10.1038/s41467-025-61841-8 (PMC12334574; doi:10.1038/s41467-025-61841-8)
Supplement: Supplementary file 1 — Supplementary Information [file 41467_2025_61841_MOESM1_ESM.pdf]

Regional heterogeneity of the blood-brain barrier

Supplementary Information:
Supplementary Figs. 1–16
Supplementary Table 1

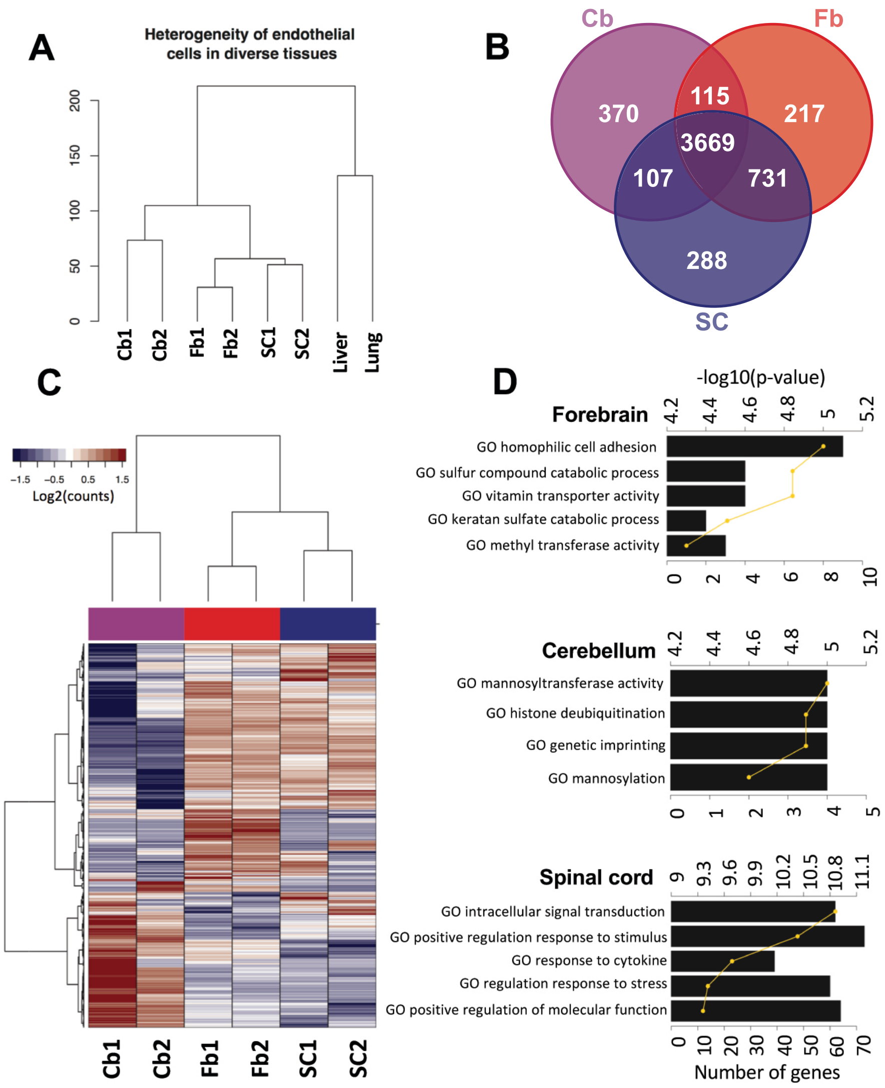

Supplementary Fig. 1: Heterogeneity of endothelial cells from brain, cerebellum and spinal cord by bulk RNA-seq. **(A)** Consensus dendrogram of RNA sequencing of endothelial cells purified from the forebrain (Fb), cerebellum (Cb), spinal cord (SC), lung or liver using 1,214 expressed and most variable genes, organized by Euclidean distance of log2 transformed FPKM values. **(B)** Venn diagrams depicting the common and unique upregulated differentially expressed genes between endothelial samples of cerebellum, forebrain, and spinal cord. Differentially expressed genes were those with FPKM > 1 among the samples compared, expression fold-change > 2, and p-value < 0.05. **(C)** Hierarchical clustering of differentially expressed genes (1759) between endothelial samples of cerebellum, forebrain, and spinal cord. Enrichment scores represent log2 transformed normalized counts of each gene. **(D)** Top 5 enriched gene ontology gene-sets obtained using upregulated differentially expressed genes. Only enriched gene-sets with p-value < 0.00005 in a hypergeometric test were considered significant. Barplots represent number of differentially expressed genes from each cell type in enriched gene-set. Yellow line depicts gene-set enrichment in a log-transformed p-value scale. Fb = forebrain, Cb = cerebellum, SC = spinal cord. Gene expression data are provided in Supplementary Data 1.

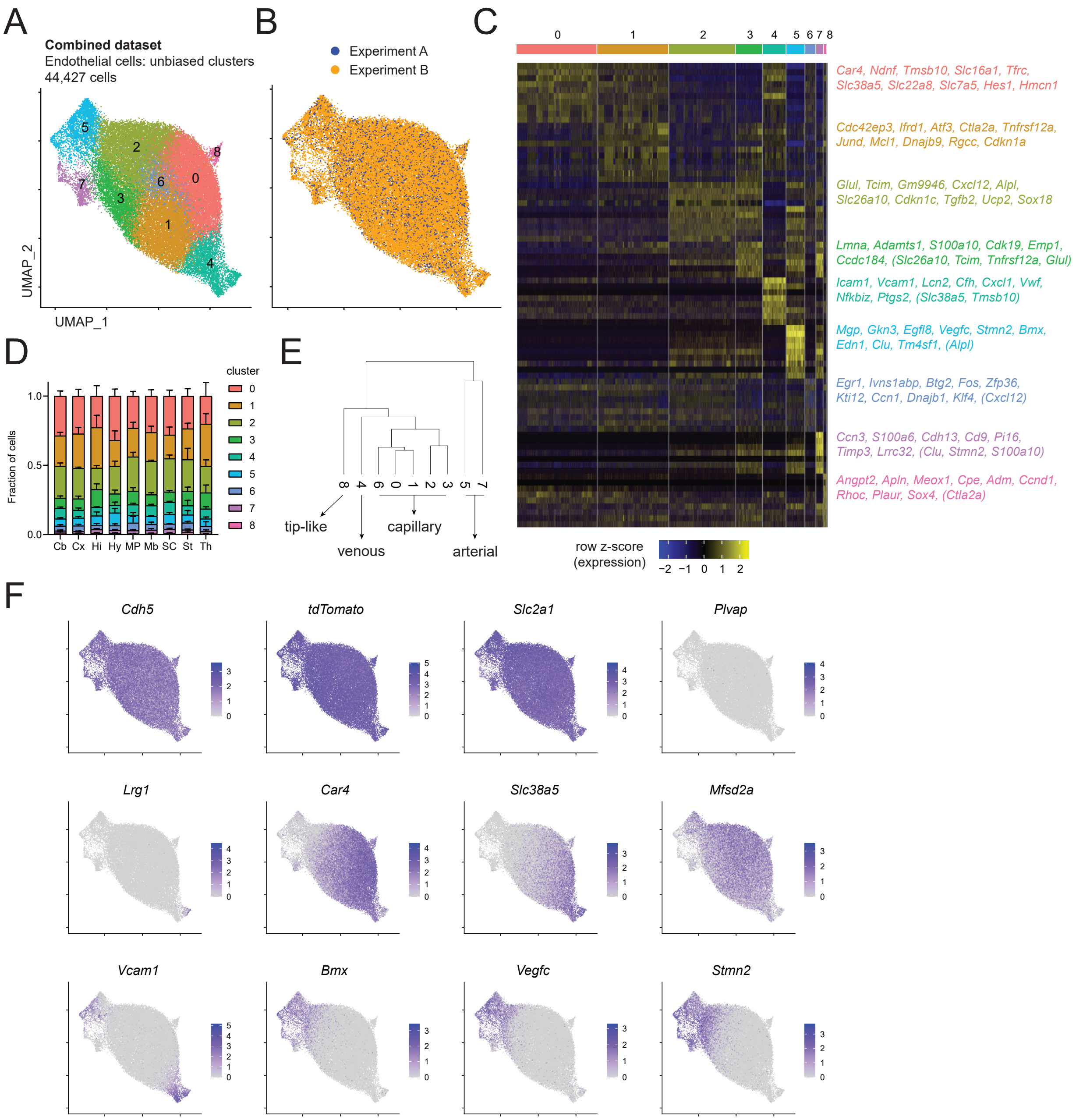

Supplementary Fig. 2: scRNA-seq analysis of intra-regional endothelial heterogeneity. (A) UMAP plot of all endothelial cells in the combined dataset (Experiments A and B). Plotted points (cells) are colored based on unbiased, graph-based clusters. **(B)** UMAP plot with points colored by experiment of origin. **(C)** Differential expression analysis comparing endothelial cell unbiased clusters. The heatmap shows genes with cluster-enriched expression. For each cluster, a list of 10 genes with the largest fold-enrichment is shown. Genes in parentheses also appear in an earlier cluster and thus are shown in an above row of the heatmap. **(D)** Fraction of endothelial cells in each unbiased cluster across brain regions. Bars indicate the mean of 3 biological replicates and error bars indicate standard deviation. One-way ANOVA, cluster 0: $F(8,18) = 1.2$, $P = 0.34$; cluster 1: $F(8,18) = 1.9$, $P = 0.12$; cluster 2: $F(8,18) = 2.0$, $P = 0.11$; cluster 3: $F(8,18) = 1.4$, $P = 0.28$; cluster 4: $F(8,18) = 1.2$, $P = 0.34$; cluster 5: $F(8,18) = 1.3$, $P = 0.31$; cluster 6: $F(8,18) = 0.45$, $P = 0.87$; cluster 7: $F(8,18) = 1.8$, $P = 0.14$; cluster 8: $F(8,18) = 1.4$, $P = 0.28$. **(E)**

Dendrogram of cluster similarity based on the first 25 Harmony components. Clusters annotated as arterial, venous, capillary, and tip-like endothelial cells are shown at bottom. **(F)** UMAP plots with points colored based on expression of indicated genes. Canonical markers of endothelial cells, BBB endothelial cells, and arteriovenous endothelial subtypes are shown: *Cdh5* and *tdTomato* (pan-endothelial), *Slc2a1* (pan-BBB endothelial), *Plvap* (non-BBB endothelial), *Lrg1* (venous-enriched), *Car4* and *Slc38a5* (capillary/venous-enriched), *Mfsd2a* (capillary-enriched), Vcam1 (venous/arterial-enriched), *Bmx*, *Vegfc*, and *Stmn2* (arterial-enriched). Color bars indicate expression (log-normalized counts). Source data are provided as a Source Data file. Gene expression data are provided in Supplementary Data 2.

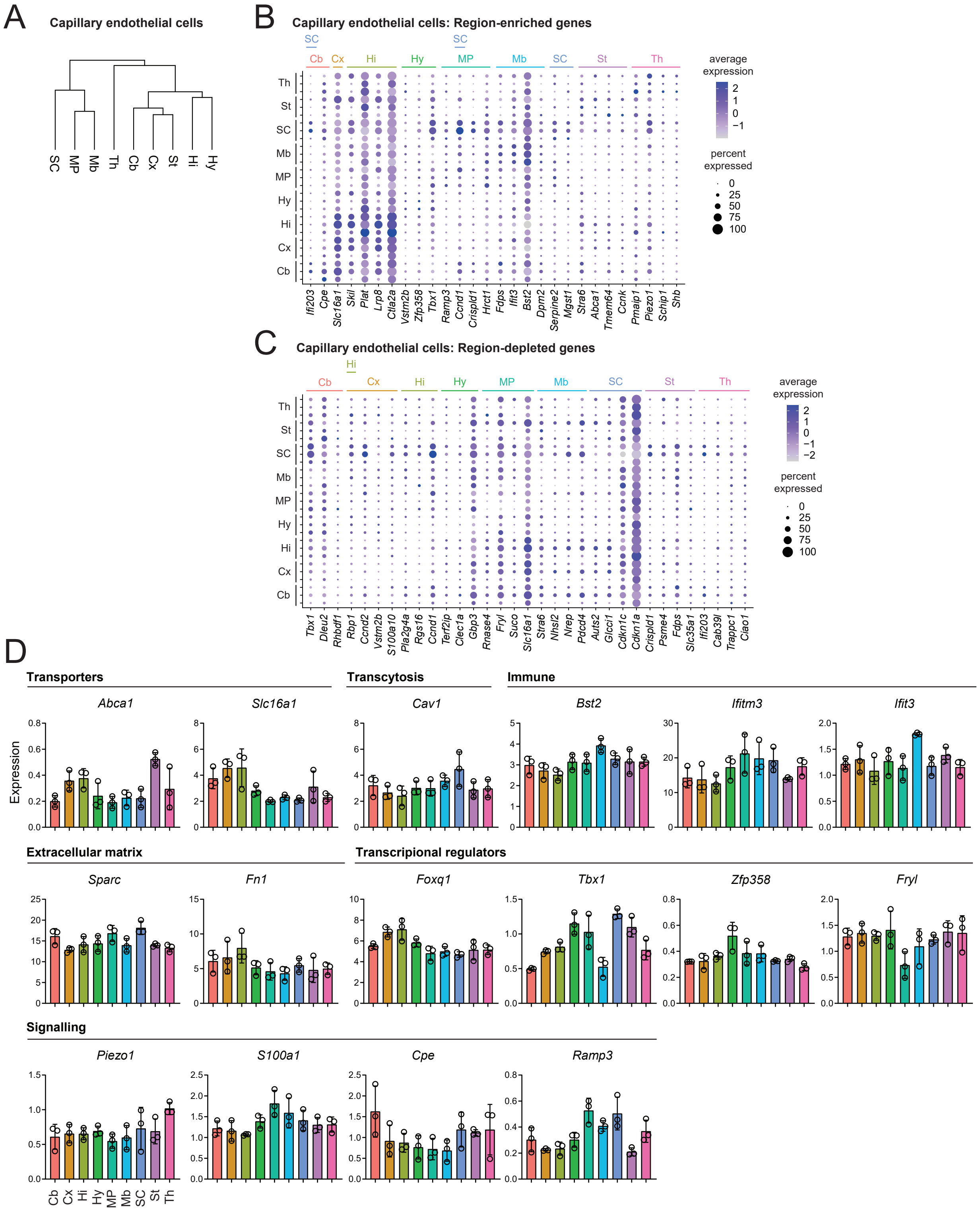

Supplementary Fig. 3: scRNA-seq analysis of inter-regional endothelial heterogeneity. (A) Dendrogram of capillary endothelial cell region similarity based on the first 25 Harmony components. **(B-C)** Dot plots of gene expression in capillary endothelial cells. Plots show genes with enriched (B) or depleted (C) expression in the region of interest compared to all other

regions. Genes shown have statistically significant enrichment or depletion (average expression > 1000 pseudobulk counts, $P < 0.05$, DESeq2 Wald test (two-sided) with Benjamini-Hochberg correction); up to 4 genes with the largest fold-enrichment/depletion are shown. Dot color indicates average expression level and size indicates the fraction of cells that express a gene. The three biological replicates are shown for each brain region. Annotations above dot plots indicate regions with statistically significant enrichment or depletion. **(D)** Expression (log-normalized counts) of indicated genes in capillary endothelial cells across brain regions. Points represent average expression in each biological replicate and error bars represent SD. Gene expression data are provided in Supplementary Data 2.

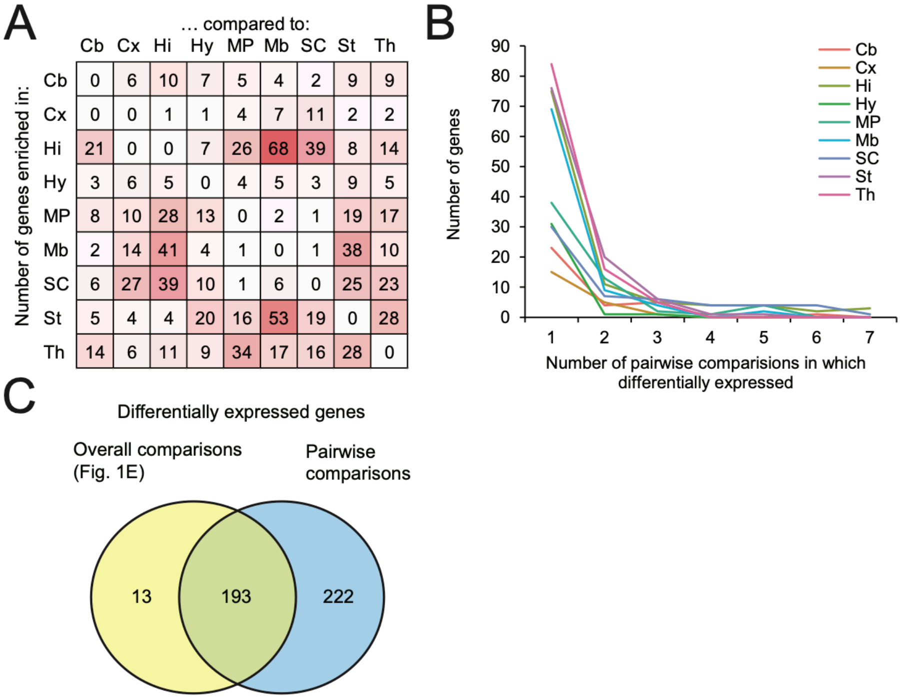

Supplementary Fig. 4: scRNA-seq pairwise differential expression analysis. (A) Number of genes enriched in each pairwise comparison. **(B)** Histogram showing the number of genes versus the number of pairwise comparisons in which a gene is differentially expressed for each brain region. **(C)** Venn diagram comparing the genes identified as differentially expressed in the overall comparison (cells in each region compared to all other cells; Fig. 1E) and genes identified as differentially expressed in at least one pairwise comparison. Complete results of pairwise differential expression analysis are provided in Supplementary Data 3.

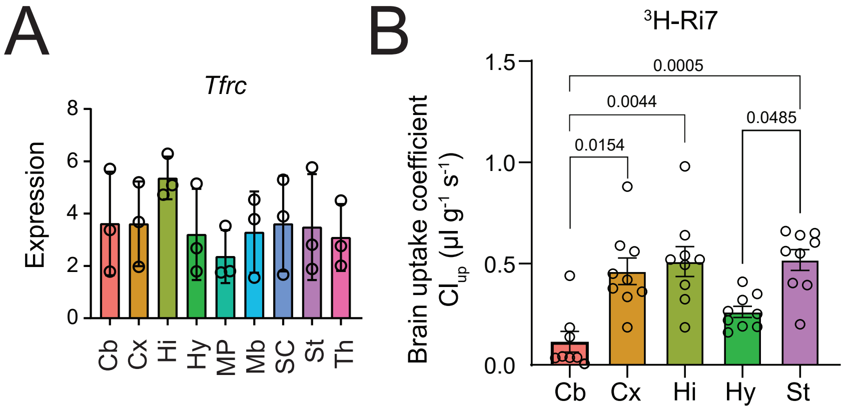

Supplementary Fig. 5: Functional validation of regional enrichment of the transferrin receptor. (A) Expression (log-normalized counts) of *Tfrc* in capillary endothelial cells across brain regions. Points represent average expression in each biological replicate and error bars represent SD. **(B)** Brain uptake of a tritiated internalizing anti-transferrin receptor antibody (^{3}H-Ri7) in five brain regions. Points represent replicate mice and error bars represent SEM. P-values: Kruskal-Wallis test followed by Dunn's post-hoc test. Source data are provided as a Source Data file. Gene expression data are provided in Supplementary Data 2.

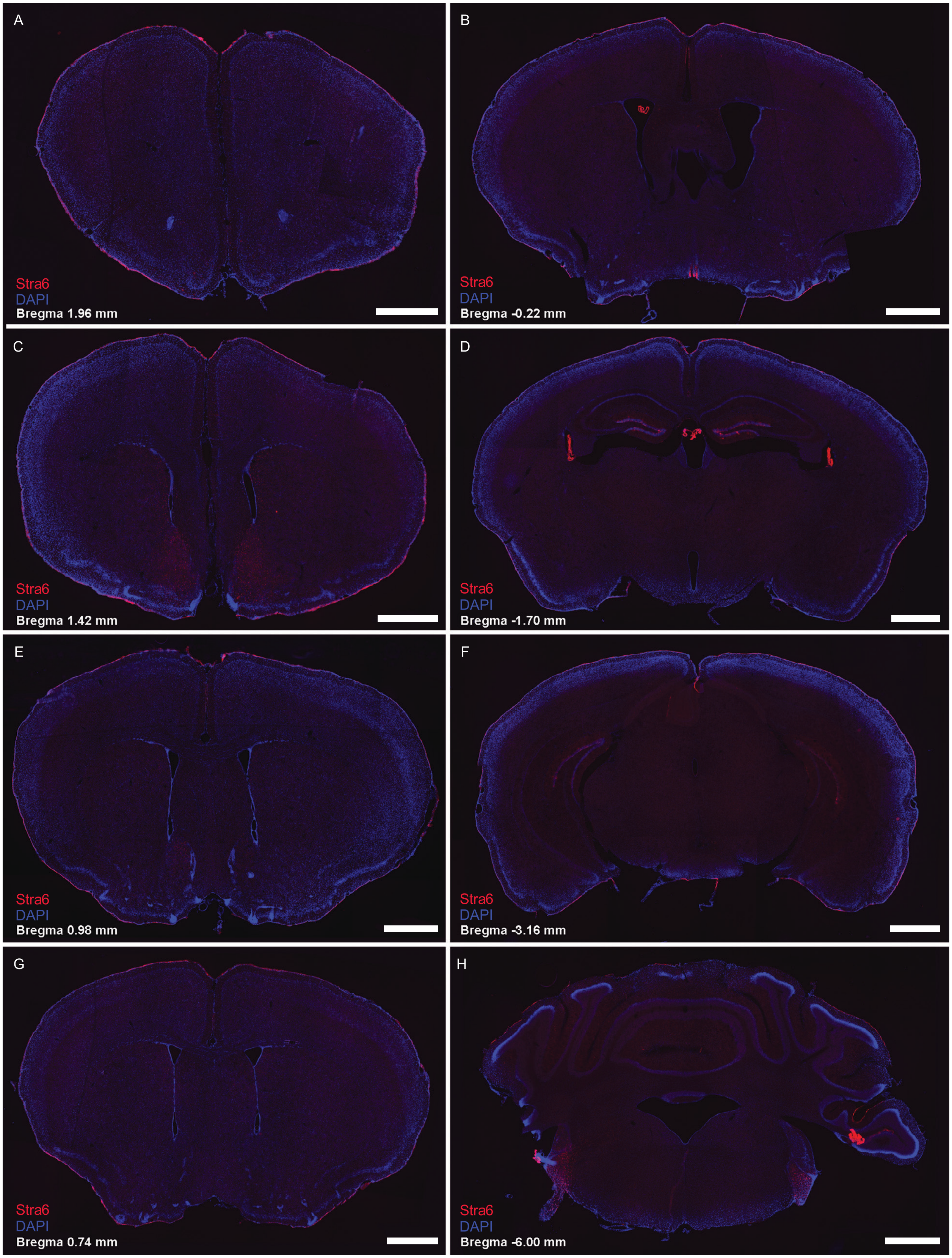
A
Stra6
DAPI
Bregma 1.96 mm
B
Stra6
DAPI
Bregma -0.22 mm
C
Stra6
DAPI
Bregma 1.42 mm
D
Stra6
DAPI
Bregma -1.70 mm
E
Stra6
DAPI
Bregma 0.98 mm
F
Stra6
DAPI
Bregma -3.16 mm
G
Stra6
DAPI
Bregma 0.74 mm
H
Stra6
DAPI
Bregma -6.00 mm

Supplementary Fig. 6: Stra6 expression across the whole brain. **(A-H)** Series of mouse brain coronal sections stained with an antibody against Stra6 (red), and with DAPI (blue). The rostrocaudal position of each section relative to bregma is (A) +1.96 mm, (B) –0.22 mm, (C) +1.42 mm, (D) –1.70 mm, (E) +0.98 mm, (F) –3.16 mm, (G) +0.74 mm, (H) –6.00 mm. Scale bars: 1 mm.

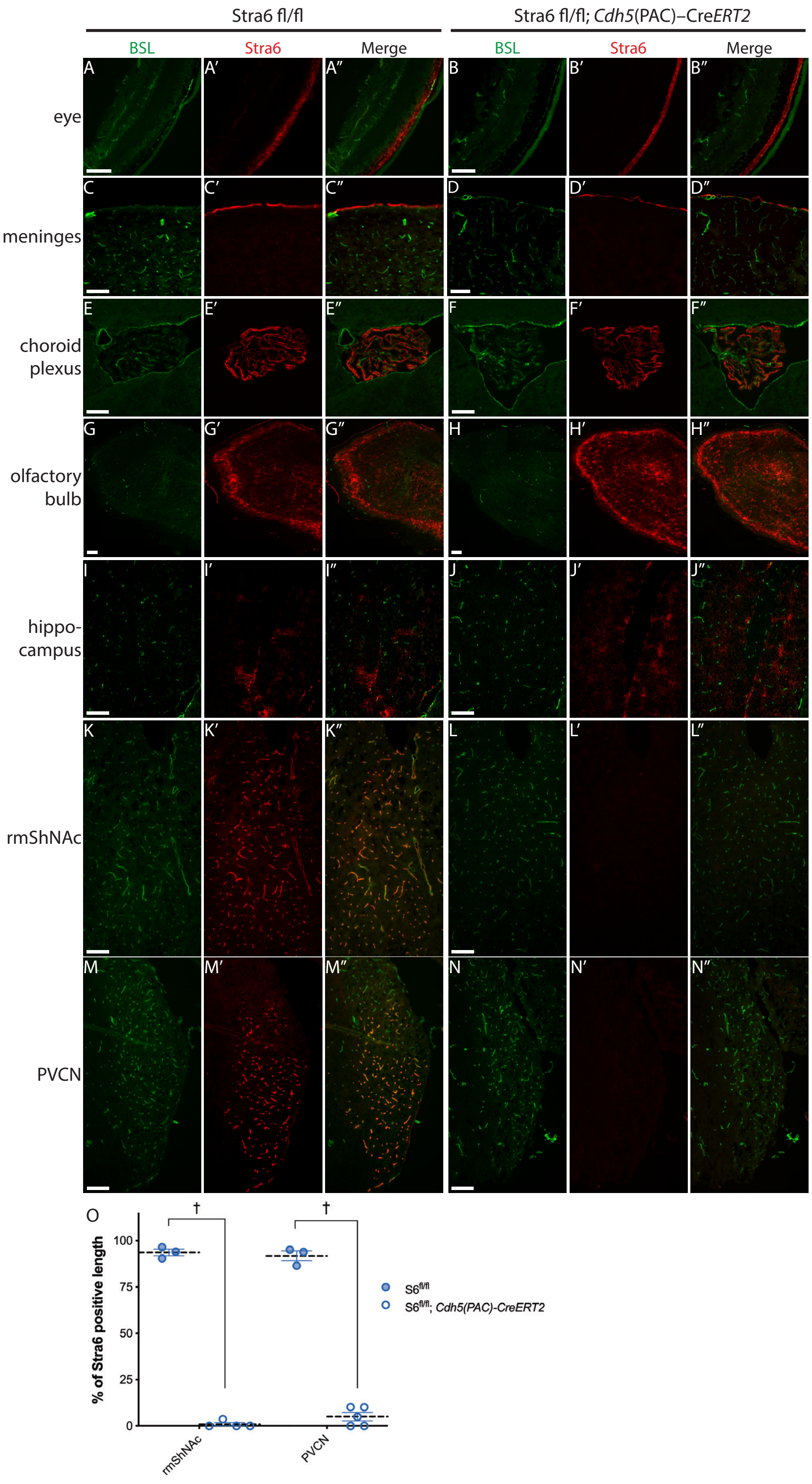
Stra6 fl/fl
Stra6 fl/fl; Cdh5(PAC)–CreERT2
BSL
Stra6
Merge
eye
meninges
choroid plexus
olfactory bulb
hippo-campus
rmShNAc
PVCN
% of Stra6 positive length
S6fl/fl
S6fl/fl; Cdh5(PAC)-CreERT2
rmShNAc
PVCN

Supplementary Fig. 7: Validation of Stra6 endothelial-specific deletion in the Stra6$^{fl/fl}$ Cdh5–CreERT2 mice. (A-N) Tissue sections from endothelial-specific Stra6 knockout mice (Stra6 fl/fl; Cdh5(PAC)-CreERT2) and littermate controls (Stra6 fl/fl) raised on conventional diet and switched to VitA-sufficient diet at weaning were stained with an antibody against Stra6 (red) and BSLI-Fluorescein (green) to label endothelial cells. Stra6 is expressed in the retinal pigment epithelium of the eye (A, B), fibroblasts in meninges (C, D), epithelial cells of the choroid plexus (E, F), neural cells in the olfactory bulb (G, H), sporadic neural cells in the hippocampus (I, J), and in the endothelial cells of the rmShNAc (K, L) and PVCN (M, N). In Stra6 endothelial-specific conditional KO mice, Stra6 expression is only deleted in endothelial cells, and its expression in other cell types is not affected. Scale bars: 100 µm for all regions except the olfactory bulb where scale bars: 200 µm. **(O)** Quantification of the percentage of Stra6 positive blood vessel length in the rmShNAc and PVCN in endothelial-specific Stra6 knockout mice and littermate controls. n=3 and 5 for rmShNAc and PVCN in Stra6 knockout mice, n=3 and 3 for rmShNAc and PVCN for littermate controls. Statistics: unpaired two-tailed t-tests; rmShNAC $^{\dagger}P = 1.7\times10^{-6}$; PVCN: $^{\dagger}P = 3.6\times10^{-7}$. Source data are provided as a Source Data file.

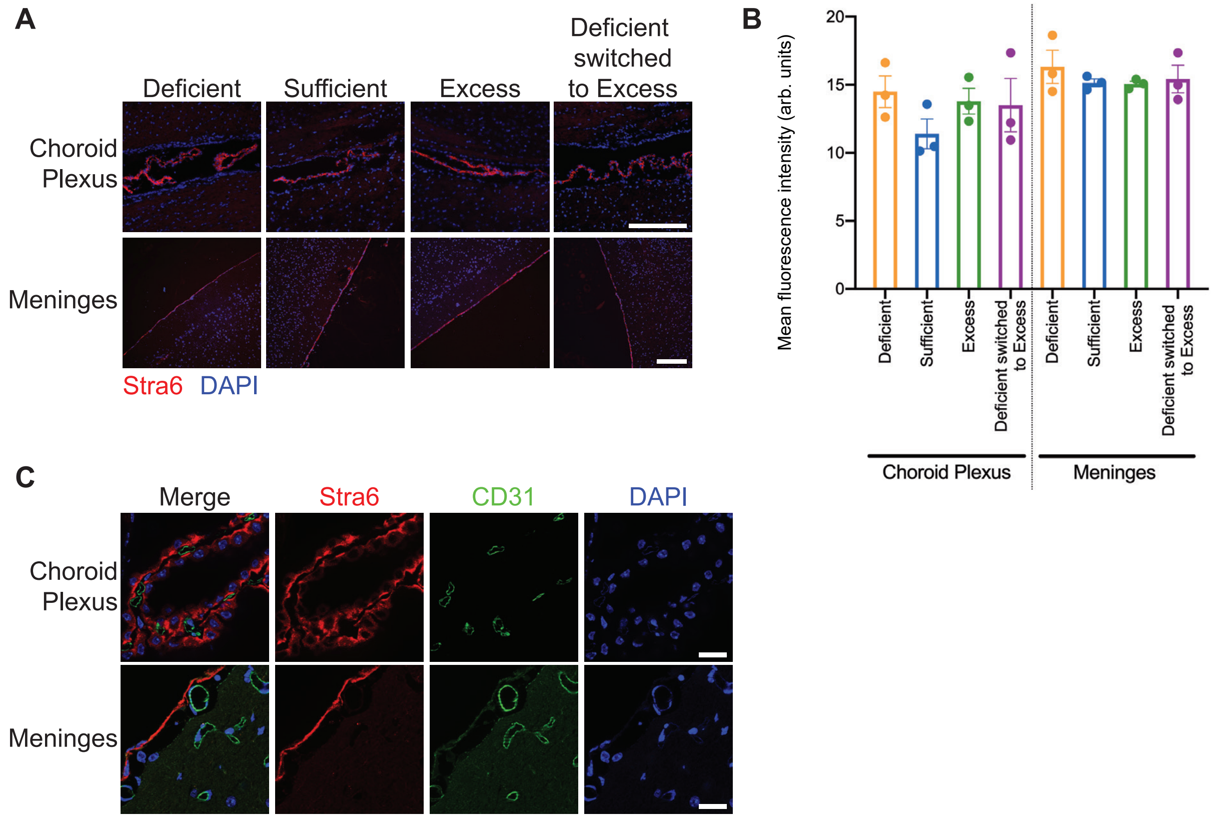

Supplementary Fig. 8: Stra6 expression at the choroid plexus and meninges is not regulated by dietary amount of Vitamin A. (A) Tissue sections of the choroid plexus and meninges from 6 week old wild type C57BL/6 mice raised on either VitA-deficient diet, VitA-sufficient diet, VitA-excess diet or raised on VitA-deficient diet then switched to VitA-excess diet for 1 week prior to analysis, were stained with antibodies against Stra6 (red) and CD31 (green). Scale bars: 200 µm. **(B)** Quantification of the Stra6 staining fluorescence intensity in the choroid plexus and meninges according to the different diet paradigms (n=3 per region and per diet). Statistics: one-way ANOVA for the different diets in each brain region; Choroid plexus P = 0.45; Meninges P = 0.69. Error bars represent SEM. **(C)** Tissue sections of the choroid plexus and meninges from C57BL/6 mice raised on conventional diet were stained with antibodies against Stra6 (red), CD31 (green) and nuclei labeled with DAPI (blue). Stra6 can be observed in choroid plexus epithelial cells, but not choroid plexus endothelial cells, and meningeal fibroblasts but not meningeal endothelial cells. Scale bars: 20 µm. Source data are provided as a Source Data file.

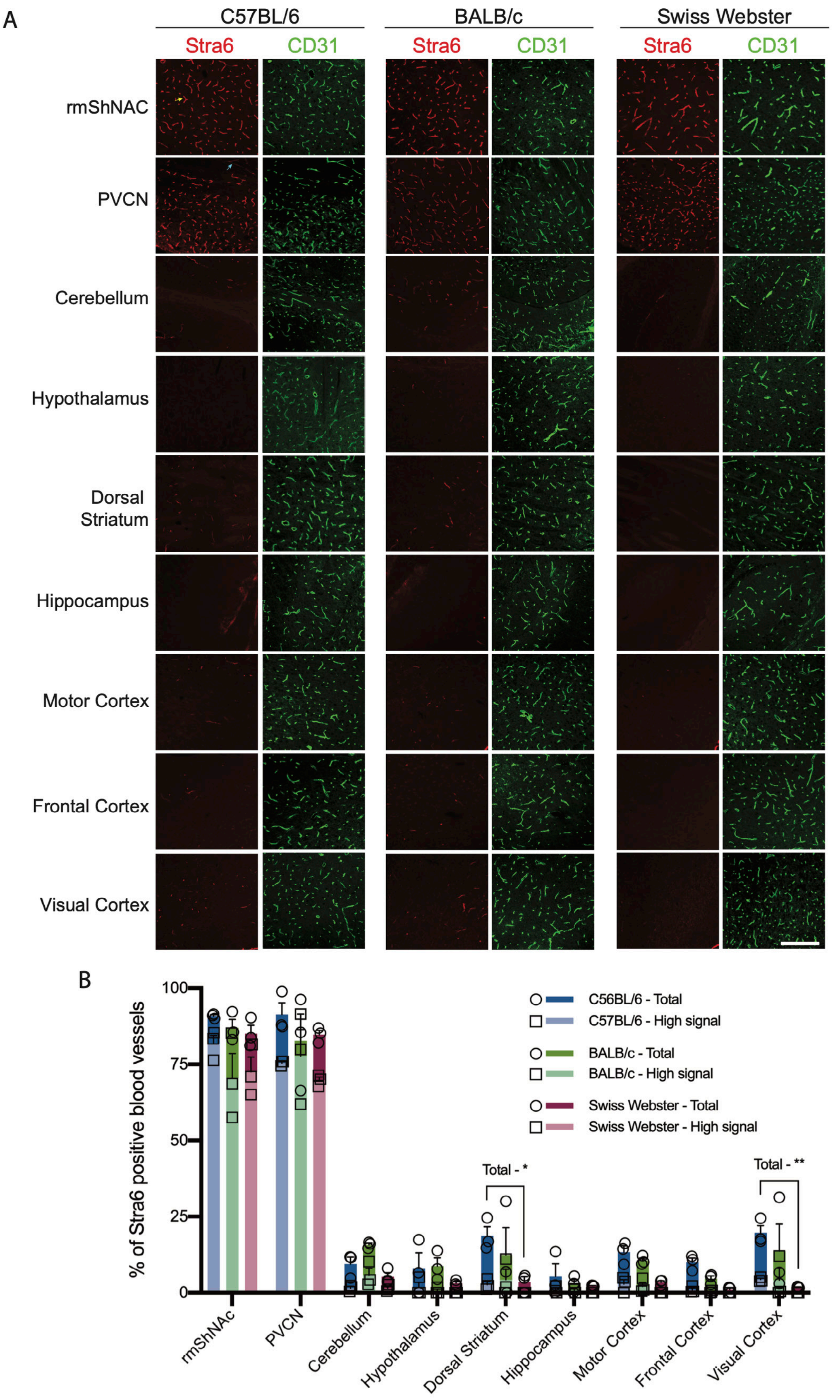
A
C57BL/6
BALB/c
Swiss Webster
Stra6
CD31
Stra6
CD31
Stra6
CD31
rmShNAC
PVCN
Cerebellum
Hypothalamus
Dorsal Striatum
Hippocampus
Motor Cortex
Frontal Cortex
Visual Cortex
B
% of Stra6 positive blood vessels
100
75
50
25
0
C56BL/6 - Total
C57BL/6 - High signal
BALB/c - Total
BALB/c - High signal
Swiss Webster - Total
Swiss Webster - High signal
Total - *
Total - **
rmShNAc
PVCN
Cerebellum
Hypothalamus
Dorsal Striatum
Hippocampus
Motor Cortex
Frontal Cortex
Visual Cortex

Supplementary Fig. 9: Stra6 expression is consistent in different mouse strains. (A) Tissue sections of the different brain regions from wild type C57BL/6, BALB/c and Swiss Webster mice raised on conventional diet stained for Stra6 (red) and CD31 (green). Scale bar: 200 µm. Yellow arrows indicate vessels with high signal, blue arrows indicate vessels with low signal **(B)** Percentage of vascular length positive for Stra6 in different brain regions according to different mouse strains: C57BL/6, BALB/c and Swiss Webster (n=3 mice per region and strain). The vascular length with both high signal and low signal was quantified for each brain region. Statistics: two-way ANOVA (region $P < 0.0001$, strain $P < 0.0001$, interaction $P < 0.0001$) followed by a Tukey's multiple comparisons test: Dorsal striatum, total signal, C57BL/6 versus Swiss Webster *$P = 0.016$; Visual cortex, total signal, C57BL/6 versus Swiss Webster **$P = 0.0018$. Error bars represent SEM. Source data are provided as a Source Data file.

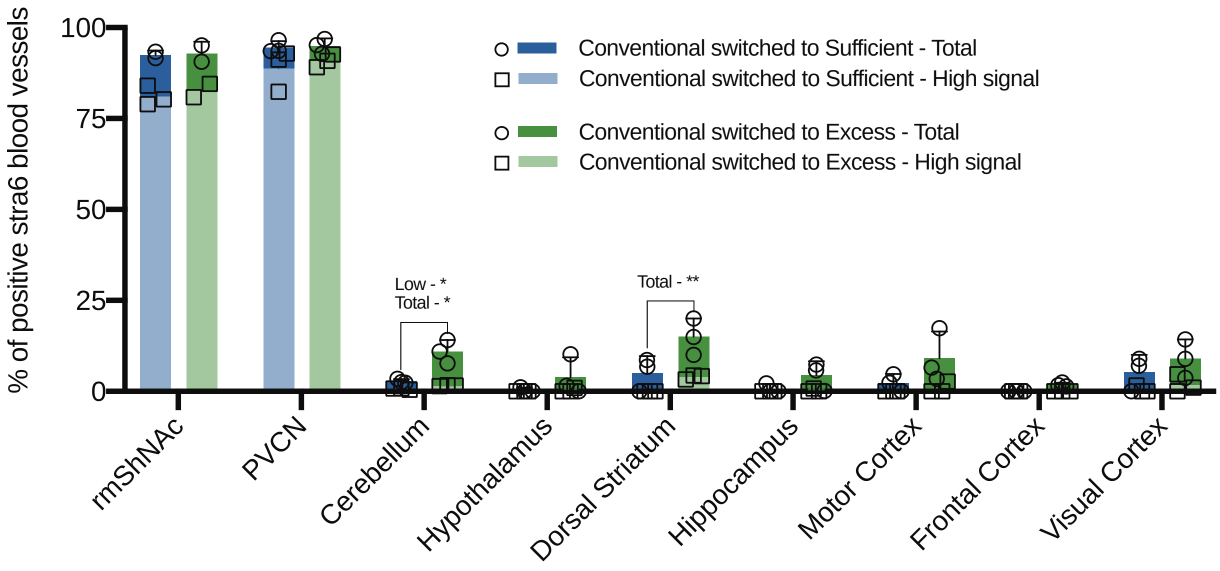

Supplementary Fig. 10: Stra6 expression at the blood-brain barrier in different brain regions for mice raised on conventional diet and switched to VitA-sufficient or VitA-excess diet at weaning. Percentage of vascular length positive for Stra6 in different brain regions according to different diet paradigm in wildtype C57BL/6 mice. Mice were kept on conventional diet and pups were transferred to either a VitA-sufficient diet or a VitA-excess diet at weaning (n=3 mice per region and diet). The vascular length with both high signal and low signal was quantified for each brain region. Statistics: two-way ANOVA (diet $P < 0.0001$, region $P < 0.0001$, interaction $P < 0.0001$) followed by a Tukey's multiple comparisons test: cerebellum, conventional switched to sufficient versus conventional switched to excess, low signal $^*P = 0.0497$, total signal $^*P = 0.019$; dorsal striatum, conventional switched to sufficient versus conventional switched to excess, total signal $^{**}P = 0.0019$. Error bars represent SEM. Source data are provided as a Source Data file.

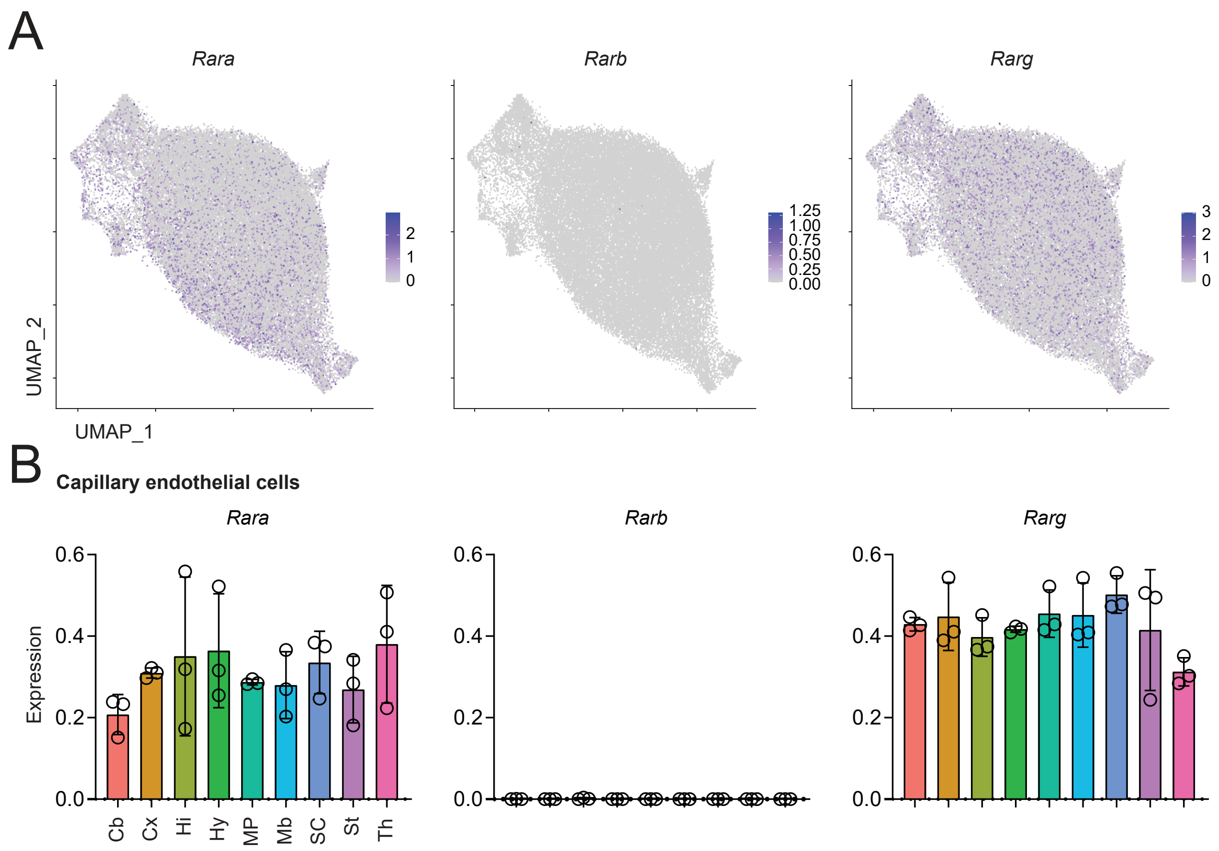

Supplementary Fig. 11: Expression of genes encoding retinoic acid receptors in scRNA-seq data. (A) UMAP plots of all endothelial cells. Points are colored based on expression of indicated genes. Color bars indicate expression (log-normalized counts). **(B)** Expression (log-normalized counts) in capillary endothelial cells across brain regions. Points represent average expression in each biological replicate and error bars represent SD. Gene expression data are provided in Supplementary Data 2.

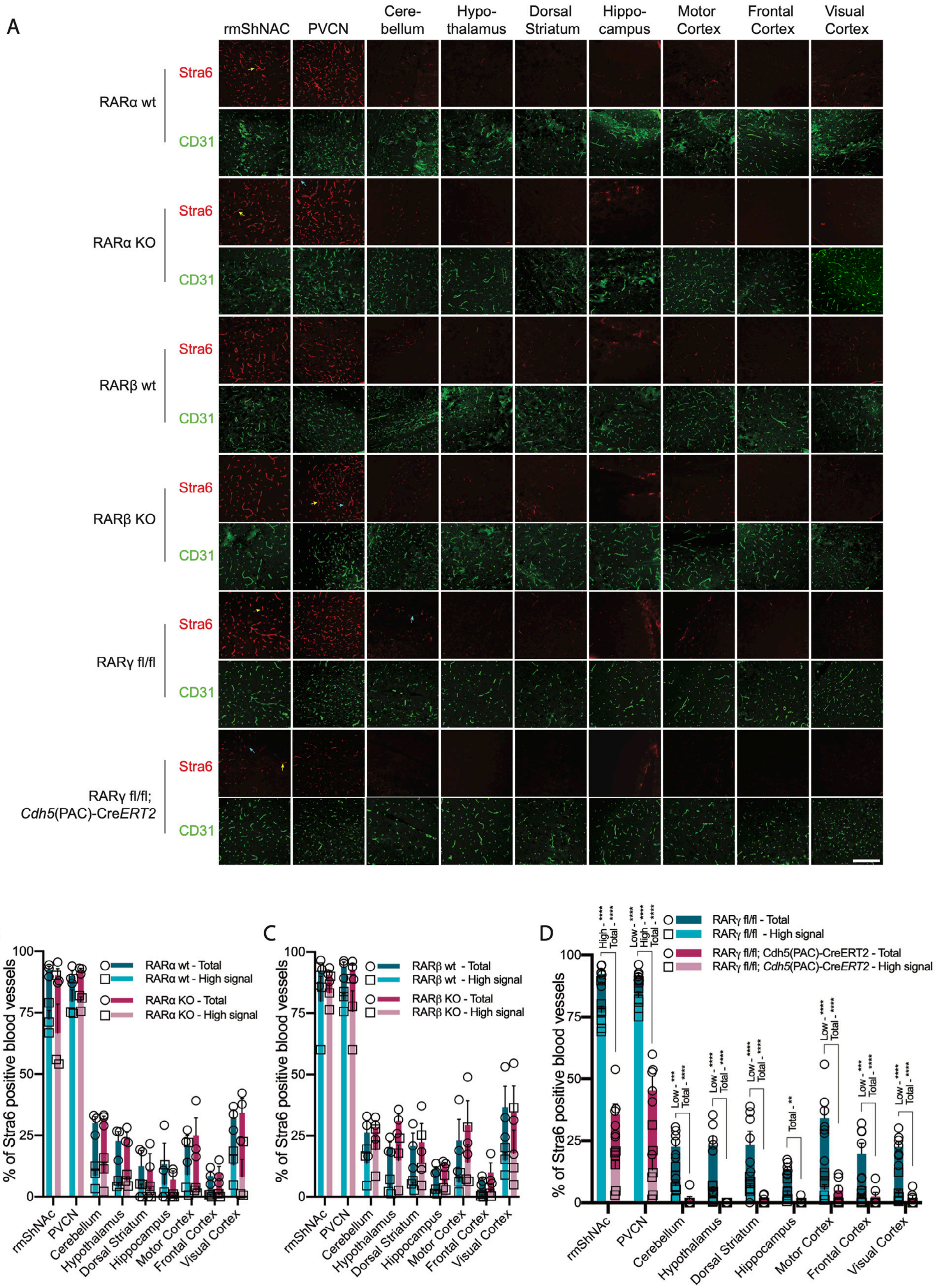

A
rmShNAC
PVCN
Cere-bellum
Hypo-thalamus
Dorsal Striatum
Hippo-campus
Motor Cortex
Frontal Cortex
Visual Cortex
RARα wt
RARα KO
RARβ wt
RARβ KO
RARγ fl/fl
RARγ fl/fl; Cdh5(PAC)-CreERT2
Stra6
CD31
B
C
D
% of Stra6 positive blood vessels
RARα wt - Total
RARα wt - High signal
RARα KO - Total
RARα KO - High signal
RARβ wt - Total
RARβ wt - High signal
RARβ KO - Total
RARβ KO - High signal
RARγ fl/fl - Total
RARγ fl/fl - High signal
RARγ fl/fl; Cdh5(PAC)-CreERT2 - Total
RARγ fl/fl; Cdh5(PAC)-CreERT2 - High signal
rmShNAc
PVCN
Cerebellum
Hypothalamus
Dorsal Striatum
Hippocampus
Motor Cortex
Frontal Cortex
Visual Cortex
0
25
50
75
100

Supplementary Fig. 12: Stra6 expression at the blood-brain barrier is regulated by endothelial RARγ. (A) Tissue sections of the different brain regions from RARα KO, RARβ KO and endothelial-specific RARγ KO mice and their littermate controls raised on conventional diet and stained against Stra6 (red) and CD31 (green). Yellow arrows indicate vessels with high signal, blue arrows indicate vessels with low signal. Scale bar: 200 µm. **(B)** Percentage of vascular length positive for Stra6 in different brain regions according to different mouse strains: RARα KO and their littermate control ($RAR\alpha^{+/+}$), **(C)** RARβ KO and their littermate controls ($RAR\beta^{+/+}$) and **(D)** endothelial-specific RARγ KO (RARγ fl/fl; Cdh5(PAC)-CreERT2) mice and their littermate controls (RARγ fl/fl). All RARγ fl/fl and RARγ fl/fl; Cdh5(PAC)-CreERT2 were both injected with tamoxifen at weaning. All mice were perfused at three months of age for analysis. For RARα and RARβ n=3 mice per region and genotype; for RARγ n=6 for mutants and n=7 for littermate controls per region. The vascular length with both high signal and low signal was quantified for each brain region. Statistics: two-way ANOVA (region $P < 0.0001$, genotype $P < 0.0001$, interaction $P < 0.0001$) followed by a Tukey's multiple comparisons test (between genotypes): rmShNAc high signal ****$P < 0.0001$, total signal ****$P < 0.0001$, PVCN low signal ****$P < 0.0001$, high signal ****$P < 0.0001$, total signal ****$P < 0.0001$, cerebellum low signal ***$P = 0.0005$, total signal ****$P < 0.0001$, hypothalamus low signal ****$P < 0.0001$, total signal ****$P < 0.0001$, dorsal striatum low signal ****$P < 0.0001$, total signal ****$P < 0.0001$, hippocampus total signal **$P = 0.0029$, motor cortex low signal ****$P < 0.0001$, total signal ****$P < 0.0001$, frontal cortex low signal ***$P = 0.0007$, total signal ****$P < 0.0001$, visual cortex low signal ****$P < 0.0001$, total signal ****$P < 0.0001$. Error bars represent SEM. Source data are provided as a Source Data file.

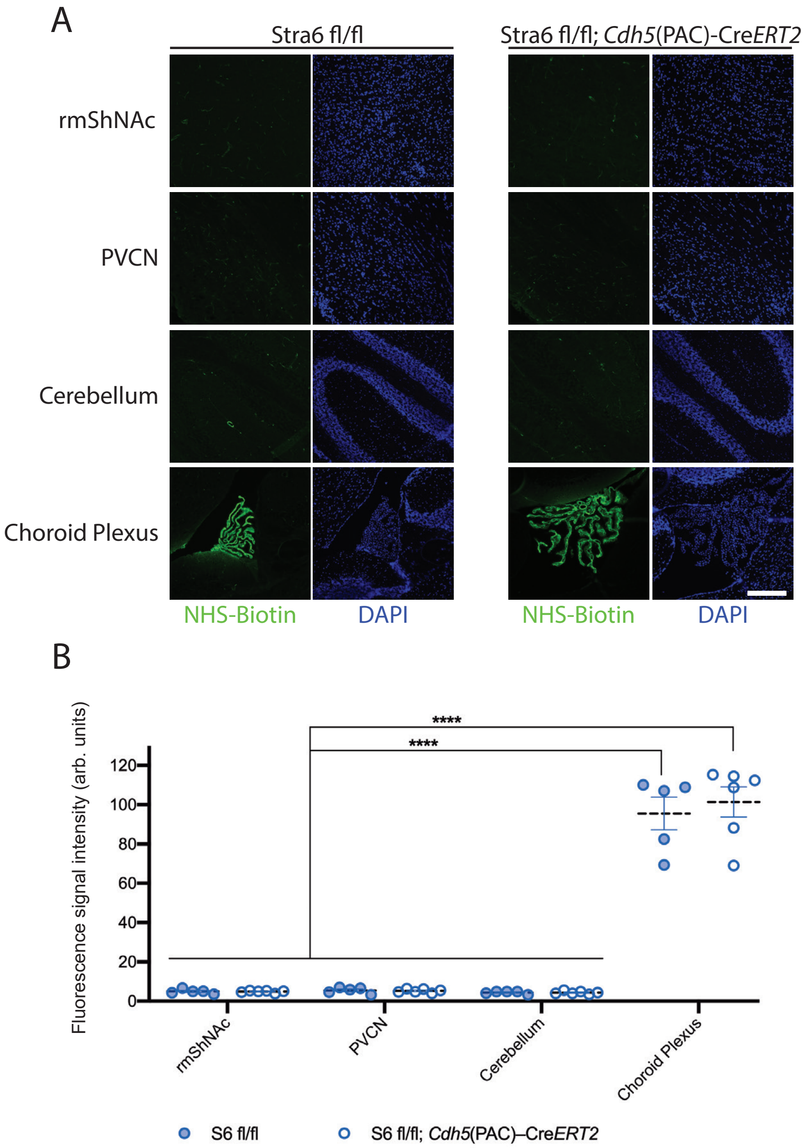

Supplementary Fig. 13: Effect of endothelial-specific Stra6 deletion on blood-brain barrier (BBB) permeability. (A) Tissues sections from endothelial-specific Stra6 knockout mice (Stra6 fl/fl; Cdh5(PAC)-CreERT2) and littermate controls (Stra6 fl/fl) raised on VitA-sufficient diet were perfused with NHS-Biotin followed by paraformaldehyde. Tissue sections were stained with a Streptavidin-Alexa-488 and parenchymal fluorescence signal intensity was quantified as an indicator of NHS-Biotin leakage in different brain regions. Scale bar: 200 μm. **(B)** Quantification of the fluorescence intensity in the rmShNAC, PVCN, Cerebellum (as an internal negative control where Stra6 is not expressed) and Choroid Plexus (as an internal positive control with leaky vessels). Conditional deletion of Stra6 from endothelial cells did not alter the permeability of the blood-brain barrier. n=6 for mutant mice, n=5 for littermate controls. Statistics: two-way ANOVA (region $P < 0.0001$, genotype $P = 0.63$, interaction $P = 0.84$) followed by a Tukey's multiple comparisons test: Stra6 fl/fl, rmShNAc versus choroid plexus ****$P < 0.0001$, PVCN versus choroid plexus ****$P < 0.0001$, cerebellum versus choroid plexus ****$P < 0.0001$; Stra6 fl/fl; Cdh5(PAC)-CreERT2 rmShNAc versus choroid plexus ****$P < 0.0001$, PVCN versus choroid plexus ****$P < 0.0001$, cerebellum versus choroid plexus ****$P < 0.0001$. Error bars represent SEM. Source data are provided as a Source Data file.

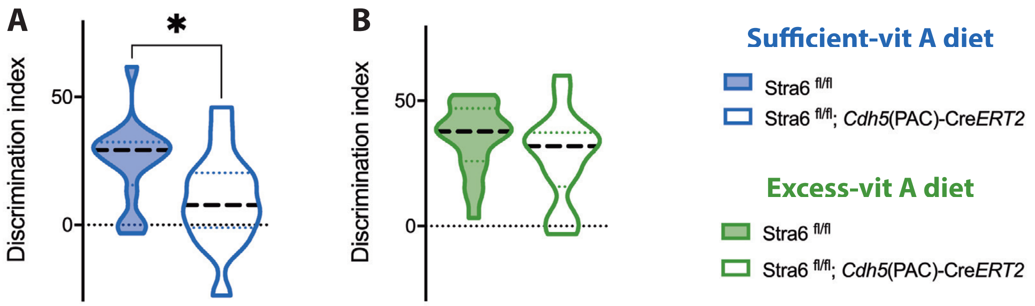

Supplementary Fig. 14: Endothelial specific Stra6 mutant mice (females) display spatial memory defect due to a reduction of retinol uptake. **(A)** Discrimination index for the novel location recognition task in female endothelial-specific Stra6 knockout mice (Stra6 f/f; *Cdh5*(PAC)-*ERT2,* n=15) and littermate controls (Stra6 f/f, n=16) raised on a VitA-sufficient diet (blue). **(B)** Discrimination index for the novel location recognition task in endothelial-specific Stra6 knockout mice (n=18) and littermate controls (n=20) raised on a VitA-excess diet (green). Statistics and P-values: unpaired two-tailed t-tests: (A) VitA-sufficient diet P = 0.022, (B) VitA-excess diet P = 0.14. Dashed lines indicate median and dotted lines indicate first and third quartiles. Source data are provided as a Source Data file.

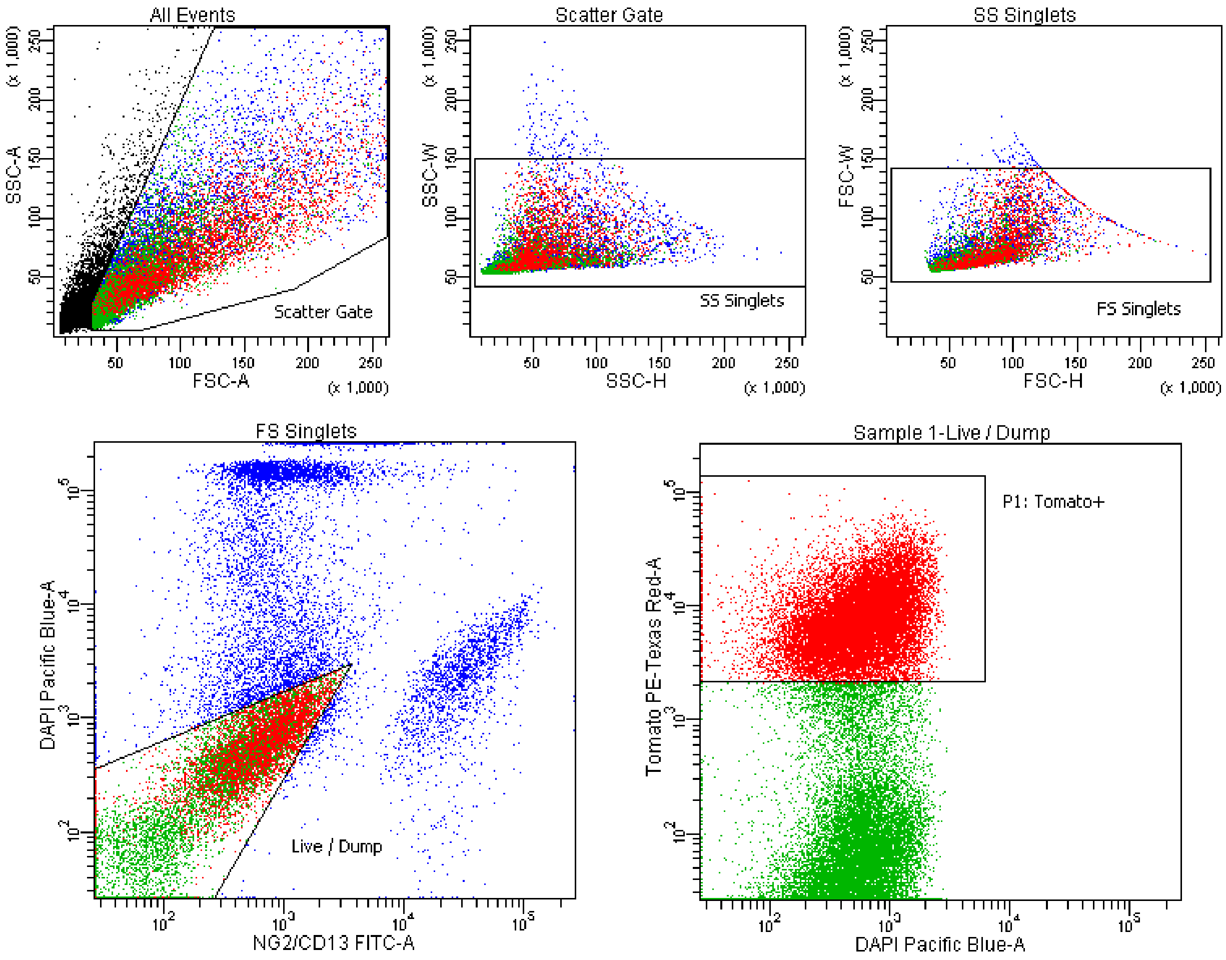

Tube: Sample 1

Population	#Events	%Parent	%Total
All Events	161,126	####	100.0
Scatter Gate	101,966	63.3	63.3
SS Singlets	100,913	99.0	62.6
FS Singlets	100,000	99.1	62.1
Live / Dump	60,786	60.8	37.7
P1: Tomato+	19,371	31.9	12.0
FS area scaling	606	0.4	0.4
SS area scaling	1,171	0.7	0.7

Supplementary Fig. 15: Purification of endothelial cells by FACS. Sample FACS plots of the purification of Cdh5-tdTomato^{+} cells used for the single-cell sequencing analysis of the brain endothelial cells from different brain regions.

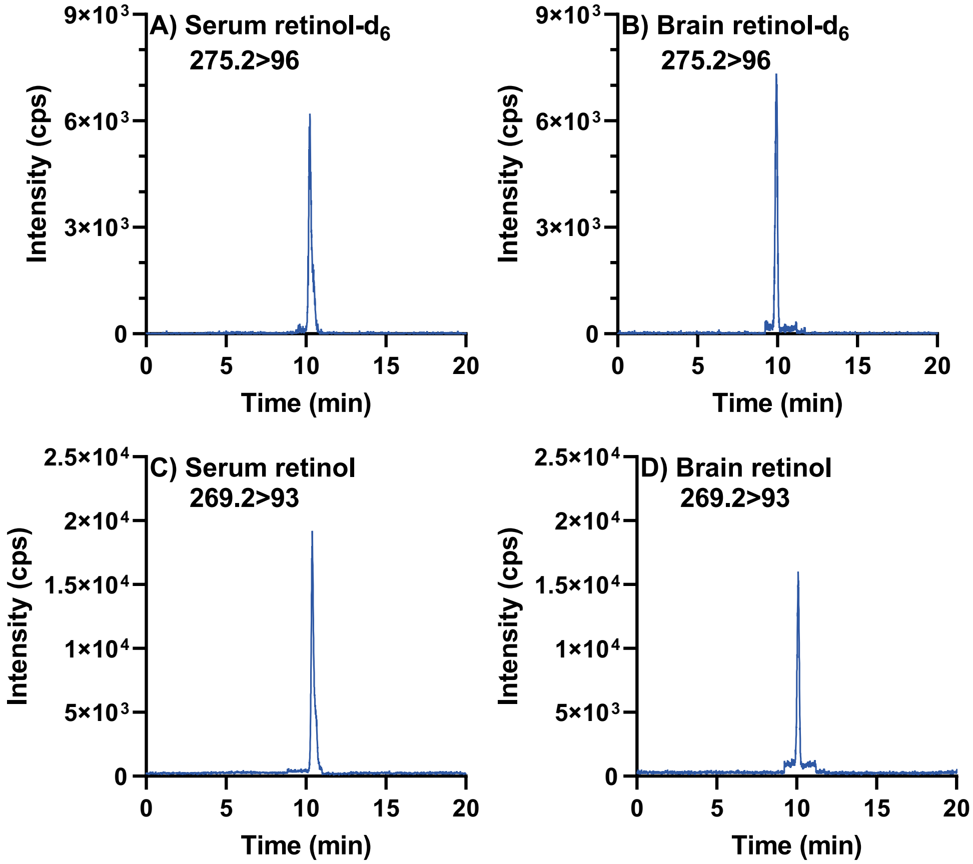

Supplementary Fig. 16: Detection of retinol by LC-MS/MS. Representative chromatograms of serum (A,C) and brain (B,D) retinol measurements from mice fed the VitA-sufficient diet. (**A,B**) Detection of the internal standard (d6 labeled retinol) in the serum and brain samples. **(C)** Detection of retinol in mouse serum. **(D)** Detection of retinol in a mouse hippocampus sample.

Supplementary Table 1. scRNA-seq experimental design and sample characteristics.

Sample	Experi-ment	Batch	Pooled mice	Est. Number of Cells	Mean Reads per Cell	Median Genes per Cell	Valid Barcodes (%)	Sequencing Saturation (%)
cerebellum-1	A	A	6	1530	42473	1312	98.0	86.0
cortex-1	A	A	3	706	93811	1156	97.9	93.2
spinal-cord-1	A	A	6	1319	51384	1411	97.8	86.1
hippocampus-1	A	B	4	532	39554	1593	97.8	80.8
hypothalamus-1	A	B	5	265	71872	1482	97.6	88.9
striatum-1	A	B	4	136	118331	1444	97.7	91.8
thalamus-1	A	B	5	232	80113	1569	97.8	89.7
medulla-pons-1	A	C	4	322	105196	1824	98.1	90.8
midbrain-1	A	C	4	885	46042	1665	98.2	81.8
hypothalamus-2	A	D	3	1284	43175	1304	97.3	87.3
medulla-pons-2	A	D	3	1049	53966	1450	97.4	88.6
striatum-2	A	D	3	375	52796	1176	96.9	90.4
thalamus-2	A	D	3	280	55797	1246	96.9	89.8
cerebellum-2	B	E	3	3381	69964	2694	97.3	67.6
spinal-cord-2	B	E	3	523	338038	3371	97.5	91.2
striatum-3	B	E	3	4457	49745	2554	97.5	59.7
cortex-2	B	F	4	6432	36242	2143	97.0	51.3
hippocampus-2	B	F	4	3474	69253	2650	97.2	65.2
medulla-pons-3	B	F	4	7061	30220	2170	97.3	56.2
cerebellum-3	B	G	3	4591	42000	1916	97.9	79.4
hippocampus-3	B	G	4	460	309555	2056	97.9	96.1
hypothalamus-3	B	G	4	2207	85105	1575	97.9	90.6
midbrain-2	B	G	4	888	176668	2069	98.1	93.7
cortex-3	B	H	4	6356	30201	1966	98.1	66.8
midbrain-3	B	H	4	6790	33759	2054	97.6	67.6
spinal-cord-3	B	H	3	2091	126988	2761	97.8	86.0
thalamus-3	B	H	4	5819	39095	2113	97.5	67.5
