# Supplementary material for: Regional heterogeneity of the blood-brain barrier
Source: Nat Commun. 2025 Aug 8;16:7332. doi: 10.1038/s41467-025-61841-8 (PMC12334574; doi:10.1038/s41467-025-61841-8)
Supplement: Supplementary file 2 — Description of Additional Supplementary Files [file 41467_2025_61841_MOESM2_ESM.pdf]

Description of Additional Supplementary Files

Supplementary Data 1:
Transcriptional profiles of endothelial cells purified from cerebellum, forebrain, and spinal cord by bulk RNA-seq. This file contains the following tabs: (**A**) gene expression matrix of FPKM values, (**B**) gene expression matrix of normalized counts, (**C-E**) differentially expressed genes (DEGs), DESeq2 test (two-sided) between (C) forebrain and cerebellum, (D) DEGs between forebrain and spinal cord, (E) DEGs between cerebellum and spinal cord, (**F**) gene-set enrichment analysis using cerebellum upregulated DEGs, (**G**) gene-set enrichment analysis using forebrain upregulated DEGs, (**H**) gene-set enrichment analysis using spinal cord upregulated DEGs.

Supplementary Data 2:
scRNA-seq differential expression analysis comparing capillary endothelial cells across brain regions. This file contains the following tabs: **(A-C)** Differential expression analysis of capillary (A), arterial (B), and venous (C) endothelial cells in each brain region (versus all other cells). For each gene, DESeq2 pseudobulk differential expression analysis results are shown: mean pseudobulk counts (baseMean), $\log_2$(fold change) and standard error (lfcSE), Wald statistic (stat), P-value (Wald test), and adjusted P-value (Benjamini-Hochberg correction). Genes with adjusted $P < 0.05$ and baseMean > 1000 are denoted as significant. The $\log_2$(fold change) in capillary endothelial cells was used to rank genes for GSEA input. **(D,E)** For each brain region, enriched and depleted gene sets from the Hallmark (D) and KEGG Pathways (E) databases are shown. For each gene set, the gene set size, GSEA enrichment score (ES), normalized enrichment score (NES), nominal P-value, false discovery rate (FDR), and familywise error rate-corrected P-value (FWER), are shown. **(F)** Summary of the number of regionally-enriched and depleted genes in capillary, arterial, and venous endothelial cells. **(G)** Pseudobulk gene expression (log-normalized counts) in capillary endothelial cells.

Supplementary Data 3:
scRNA-seq pairwise differential expression analysis of capillary endothelial cells across brain regions. This file contains the following tabs: **(A-I)** Genes enriched in each brain region compared to each other brain region are shown. Genes were considered enriched if they met the following pseudobulk differential expression analysis criteria: adjusted $P < 0.05$ and baseMean > 1000. **(J)** Number of genes enriched in each pairwise comparison. **(K)** Number of pairwise comparisons in which each gene was enriched. **(L)** Lists of genes (i) appearing in at least one pairwise comparison, but not in the overall differential expression analysis, (ii) appearing in the overall differential expression analysis but in no pairwise comparisons, and (iii) appearing in both the pairwise and overall differential expression analyses.
