# Supplementary material for: Regional heterogeneity of the blood-brain barrier
Source: Nat Commun. 2025 Aug 8;16:7332. doi: 10.1038/s41467-025-61841-8 (PMC12334574; doi:10.1038/s41467-025-61841-8)
Supplement: Supplementary file 6 — Reporting Summary [file 41467_2025_61841_MOESM6_ESM.pdf]

Corresponding author(s): Richard Daneman
Last updated by author(s): Jun 11, 2025

Reporting Summary

Nature Portfolio wishes to improve the reproducibility of the work that we publish. This form provides structure for consistency and transparency in reporting. For further information on Nature Portfolio policies, see our Editorial Policies and the Editorial Policy Checklist.

Statistics

For all statistical analyses, confirm that the following items are present in the figure legend, table legend, main text, or Methods section.

n/a	Confirmed	
☐	☒	The exact sample size (*n*) for each experimental group/condition, given as a discrete number and unit of measurement
☐	☒	A statement on whether measurements were taken from distinct samples or whether the same sample was measured repeatedly
☐	☒	The statistical test(s) used AND whether they are one- or two-sided<br>*Only common tests should be described solely by name; describe more complex techniques in the Methods section.*
☐	☒	A description of all covariates tested
☐	☒	A description of any assumptions or corrections, such as tests of normality and adjustment for multiple comparisons
☐	☒	A full description of the statistical parameters including central tendency (e.g. means) or other basic estimates (e.g. regression coefficient) AND variation (e.g. standard deviation) or associated estimates of uncertainty (e.g. confidence intervals)
☐	☒	For null hypothesis testing, the test statistic (e.g. *F*, *t*, *r*) with confidence intervals, effect sizes, degrees of freedom and *P* value noted<br>*Give P values as exact values whenever suitable.*
☒	☐	For Bayesian analysis, information on the choice of priors and Markov chain Monte Carlo settings
☐	☒	For hierarchical and complex designs, identification of the appropriate level for tests and full reporting of outcomes
☐	☒	Estimates of effect sizes (e.g. Cohen's *d*, Pearson's *r*), indicating how they were calculated

Our web collection on statistics for biologists contains articles on many of the points above.

Software and code

Policy information about availability of computer code

Data collection	Sedacom software (Panlab),<br>Bulk RNA-seq dataset: Sequencing libraries were generated from total RNA samples extracted from cerebellum, forebrain, spinal cord, liver, and lung endothelial cells. bcl2fastq (v1.8.4) was used to convert base calls and generate *.fastq files in the HiSeq 2000 sequencer.<br>Single-cell RNA-seq: Single cell gene expression V2 and V3 kits (10X Genomics) were used. Purified endothelial cells were processed according to 10X Genomics manufacturer instructions and sequenced on HiSeq4000 and NovaSeq 6000 sequencers (Illumina).
Data analysis	Microsoft Excel version 16.49, Graphpad Prism 9;<br>Bulk RNA-seq dataset: The quality of raw reads in the *.fastq files was determined using FASTQC and reads were mapped to the mm10 mouse reference genome (GENCODE M14) using TopHat (v2.1.0) with default parameters. Aligned reads were assembled using Cufflinks (v2.2.1) with standard parameters. A combined annotation file obtained by merging GENCODE M14 (www.gencodegenes.org) with NCBI lncRNA definitions (downloaded on 06/07/2017) was used. Aligned reads were converted to counts using HTSeq (0.6.0) using default parameters. Downstream data analysis were performed in R. Differential gene expression analysis was performed using DESeq2 (1.10.1). A hypergeometric test (phyper function) was performed to determine gene set enrichment using mouse gene ontology terms (GO) downloaded from http://bioinf.wehi.edu.au/software/MSigDB/.<br>Single Cell RNA-seq: CellRanger v6.0.1, Seurat v4.0.5.9003, SCTransform v0.3.2, Harmony v0.1.0, DESeq2 v1.38.3, and GSEA v4.1.0.<br>Imaging: AxioVis4O V4.8.2.0, Image J 1.52a

For manuscripts utilizing custom algorithms or software that are central to the research but not yet described in published literature, software must be made available to editors and reviewers. We strongly encourage code deposition in a community repository (e.g. GitHub). See the Nature Portfolio guidelines for submitting code & software for further information.

Data

Policy information about availability of data

All manuscripts must include a data availability statement. This statement should provide the following information, where applicable:

- Accession codes, unique identifiers, or web links for publicly available datasets
- A description of any restrictions on data availability
- For clinical datasets or third party data, please ensure that the statement adheres to our policy

Bulk RNA-seq and scRNA-seq files are available through the Gene Expression Omnibus repository with accession codes: GSE171105 and GSE165457, respectively.

Research involving human participants, their data, or biological material

Policy information about studies with human participants or human data. See also policy information about sex, gender (identity/presentation), and sexual orientation and race, ethnicity and racism.

Reporting on sex and gender	Not applicable, no human participants.
Reporting on race, ethnicity, or other socially relevant groupings	Not applicable, no human participants.
Population characteristics	Not applicable, no human participants.
Recruitment	Not applicable, no human participants.
Ethics oversight	Not applicable, no human participants.

Note that full information on the approval of the study protocol must also be provided in the manuscript.

Field-specific reporting

Please select the one below that is the best fit for your research. If you are not sure, read the appropriate sections before making your selection.

☒ Life sciences ☐ Behavioural & social sciences ☐ Ecological, evolutionary & environmental sciences

For a reference copy of the document with all sections, see nature.com/documents/nr-reporting-summary-flat.pdf

Life sciences study design

All studies must disclose on these points even when the disclosure is negative.

Sample size	Statistical methods were not used to predetermine sample sizes. Sample sizes were selected based on variability of the measurement and values of difference between conditions.
Data exclusions	For in situ perfusion of the tritiated anti-transferrin receptor antibody, the cerebellum from one mouse was excluded due to poor perfusion. No other data were excluded from the analysis.
Replication	Bulk RNA-seq was obtained from two biological replicates of endothelial cells isolated from cerebellum, forebrain, and spinal cord. One sample from endothelial cells isolated from liver and lung were used for comparison. For single cell RNA-Seq, Cerebellum, cortex, hippocampus, hypothalamus, medulla/pons, spinal cord, striatum and thalamus were dissected from 3-6 mice and pooled to form each biological replicate. Two rounds of isolation were performed independently by different experimenters, with 1-2 biological replicates per round, for a total of 3 biological replicates per brain region. For experiments examining the regulation of Stra6 expression by dietary amount of Vitamin A n=3 per brain region and diet. For the experiments showing that the endothelial RARgamma regulates Stra6 expression at the BBB; RARg fl/fl; Cdh5(PAC)-ERT2 n=6 mice and RARg fl/fl, n=7. For the discrimination index for the novel location recognition task in endothelial-specific Stra6 knockout mice (Stra6 f/f; Cdh5(PAC)-ERT2, n=23) and littermate controls (Stra6 f/f, n=20) raised on a VitA-sufficient diet. For the latency to fall in endothelial-specific Stra6 knockout (n=16) and littermate control mice (n=10) raised on VitA-sufficient diet. For the discrimination index for the novel object recognition task in in endothelial-specific Stra6 knockout mice (n=9) and littermate controls (n=9) fed on VitA-sufficient diet. For the discrimination index for the novel location recognition task in endothelial-specific Stra6 knockout mice (n=21) and littermate controls (n=24) raised on a VitA-excess diet. For the latency to fall in endothelial-specific Stra6 knockout (n=7) and littermate control mice (n=11) raised on VitA-excess diet. For the validation of Stra6 endothelial-specific deletion of Stra6 fl/fl Cdh5–CreERT2 mice; n=4 and 5 for rmShNAc and PVCN mutant mice, n=3 and 4 for rmShNAc and PVCN for littermate controls. For the quantification of the Stra6 staining fluorescence intensity in the choroid plexus and meninges according to the different diet paradigms (n=3 per region and per diet). For the validation of Stra6 regional specialization in different mice strains: n=3 per brain region and mice strain. For the Stra6 quantification at the blood-brain barrier of different brain regions in C56BL/6 mice raised on conventional diet and switched to either VitA-sufficient or VitA-excess diet at weaning: n=3 per region and per diet. For the demonstration that Stra6 expression is regulated by RARg and not RARa and RARb at the blood-brain barrier: n=3 per brain region and genotype for RARa and RARb KO and wt mice; n=6 per brain regions for Rarg mutants and n=7 fper brain regions for RARγ littermate controls. Effect of endothelial-specific Stra6 deletion on the blood-brain barrier (BBB) permeability n=6 for mutant mice, n=5 for littermate controls. In situ perfusion of tritiated anti-transferrin receptor antibody: n=9 mice.

Randomization	Animals were assigned to various experimental groups randomly.
Blinding	Investigators were blinded to groups during data collection and analysis.

Reporting for specific materials, systems and methods

We require information from authors about some types of materials, experimental systems and methods used in many studies. Here, indicate whether each material, system or method listed is relevant to your study. If you are not sure if a list item applies to your research, read the appropriate section before selecting a response.

Materials & experimental systems

n/a	Involved in the study
☐	☒ Antibodies
☒	☐ Eukaryotic cell lines
☒	☐ Palaeontology and archaeology
☐	☒ Animals and other organisms
☒	☐ Clinical data
☒	☐ Dual use research of concern
☒	☐ Plants

Methods

n/a	Involved in the study
☒	☐ ChIP-seq
☒	☐ Flow cytometry
☒	☐ MRI-based neuroimaging

Antibodies

Antibodies used	Primary antibodies: Rabbit anti-Stra6, 1:1000, Abgent cat# AP9433b; Rat anti-CD31, 1:1000, BD cat# 553370) and secondary antibodies (Goat anti-Rabbit IgG and Goat anti-Rat IgG Cross-Adsorbed, Life technologies).
Validation	We validated Rabbit anti-Stra6 antibody by staining tissue from Stra6 complete KO mice and endothelial-specific Stra6 KO mice. We also cross-adsorbed Rabbit anti-Stra6 with Stra6 complete KO mice tissue to remove antibodies non-specific to Stra6. All other antibodies are well characterized by the manufacturers or by our group.

Animals and other research organisms

Policy information about studies involving animals; ARRIVE guidelines recommended for reporting animal research, and Sex and Gender in Research

Laboratory animals	Tie2GFP mice (Jax stock# 003658), Cg-Gt(ROSA)26Sor<tm9(CAG-tdTomato)Hze mice (Jax stock# 007909), RARalpha knockout (Jax stock# 023845) and RARbeta knockout (Jax stock# 022999) mice were acquired from Jackson labs. Stra6 floxed mice (MGI: 5532504) and RARgamma floxed mice (MGI:2386111) were generously provided by Norbert B. Ghyselinck. Tg (Cdh5-cre/ERT2) (MGI:3848982) were kindly given by Ralph H. Adams. In order to assess the impact of dietary VitA uptake on Stra6 expression at the BBB C57BL/6 wild-type mice were analysis at 6 weeks of age. In order to identify which RAR nuclear receptors regulate Stra6 expression at the BBB, RARalpha heterozygous breeding pairs, RARbeta heterozygous breeding pairs and RARgamma fl/fl; Cdh5-cre/ERT2/+ mice crossed with RARgamma fl/fl breeding pairs were analysed at 8 weeks of age. In addition, RARgamma fl/fl; Cdh5-cre/ERT2/+ mice crossed with RARgamma fl/fl were mated on standard rodent chow and at E14 dams were transferred to a VitA-deficient diet, and transferred to the VitA-excess diet at 5 weeks of age and analyzed at 6 weeks of age. Females and males mice were analyzed for all experiments done to examine the regulation of Stra6 expression at the BBB. For behavioral experiments, endogenous retinols measurements, serum RBP4 measurements, BBB permeability assay and validation of the Stra6 endothelial specific KO mice, breeding pairs of Stra6 endothelial specific inducible conditional knockout mice were kept on standard rodent chow and pups were transfered to either a VitA-sufficient diet or a VitA-excess diet at weaning and analyzed at 3 months of age.
Wild animals	Not applicable
Reporting on sex	All mice used for bulk RNA-seq and scRNA-seq were male. Discrimination index assay was performed in both male and female mice, and these data are included in the manuscript.
Field-collected samples	Not applicable
Ethics oversight	All experiments were performed under University of California, San Diego or Université Laval IACUC approval.<br>University of California, San Diego IACUC protocol number: S14044<br>Université Laval: CPAUL, 20-625

Note that full information on the approval of the study protocol must also be provided in the manuscript.

Plants

Seed stocks	Not applicable
Novel plant genotypes	Not applicable
Authentication	Not applicable
